# Structural Basis of GABA_B_ Receptor Activation during Evolution

**DOI:** 10.1002/advs.202509440

**Published:** 2025-07-12

**Authors:** Guofei Hou, Shenglan Zhang, Cangsong Shen, Suyu Ji, Binqian Zou, Chanjuan Xu, Liang Li, Dandan Shen, Jiayin Liang, Haidi Chen, Philippe Rondard, Cheng Deng, Jun He, Yan Zhang, Jianfeng Liu

**Affiliations:** ^1^ Cellular Signaling laboratory International Research Center for Sensory Biology and Technology of MOST Key Laboratory of Molecular Biophysics of MOE School of Life Science and Technology Huazhong University of Science and Technology Wuhan 430074 China; ^2^ Bioland Laboratory (Guangzhou Regenerative Medicine and Health Guangdong Laboratory) Guangzhou 510005 China; ^3^ Hubei Jiangxia Laboratory Wuhan Hubei 430200 China; ^4^ Department of Biophysics and Department of Pathology of Sir Run Shaw Hospital Zhejiang University School of Medicine Hangzhou 310058 China; ^5^ CAS Key Laboratory of Regenerative Biology Guangdong Provincial Key Laboratory of Stem Cell and Regenerative Medicine GIBH‐HKU Guangdong‐Hong Kong Stem Cell and Regenerative Medicine Research Centre GIBH‐CUHK Joint Research Laboratory on Stem Cell and Regenerative Medicine Guangzhou Institutes of Biomedicine and Health Chinese Academy of Sciences Guangzhou 510530 China; ^6^ Department of Respiratory and Critical Care Medicine Center for High Altitude Medicine Institutes for Systems Genetics National Clinical Research Center for Geriatrics West China Hospital Sichuan University Chengdu 610213 China; ^7^ Institut de Génomique Fonctionnelle Univ. Montpellier CNRS INSERM Montpellier 34094 France; ^8^ Liangzhu Laboratory Zhejiang University Hangzhou 311121 China; ^9^ Center for Structural Pharmacology and Therapeutics Development Sir Run Run Shaw Hospital Zhejiang University School of Medicine Hangzhou 310016 China

**Keywords:** cryo‐EM, evolution, GABA, GABA_B_ receptor, GPCR (G protein‐coupled receptor)

## Abstract

GABA_B_ receptor is a Class C G protein‐coupled receptor (GPCR) for γ‐aminobutyric acid (GABA), the principal inhibitory neurotransmitter. It forms an obligatory heterodimer consisting of two subunits, GB1 and GB2. Whether the activation mechanism of the GABA_B_ receptor is conserved during evolution remains unknown. Here, the cryogenic electron microscopy (cryo‐EM) structures of the *drosophila* GABA_B_ receptor in both antagonist‐bound inactive state and GABA‐bound active state in complex with G_i_ protein are reported. The *drosophila* GABA_B_ receptor exhibits an asymmetric activation, mirroring its human homolog. However, a larger inactive interface prevents *drosophila* GABA_B_ receptor constitutive activity. Four key residues, which are not conserved in *drosophila* GABA_B_ receptor, are responsible for the activity of the positive allosteric modulator in its human homolog. Whereas the intracellular loop 2 of *drosophila* GB2 (dGB2) is less involved, the ordered C terminus of dGB2 and its corresponding region in its human homolog are required for G protein coupling. These evolutionary variations provide a complete understanding of the activation mechanism of the GABA_B_ receptor and new insights for future development of allosteric modulators for medication and insecticides.

## Graphical abstract

This study explores the structural and functional mechanisms of the *drosophila* GABA_B_ receptor, a key role in neurotransmission. Using cryo‐EM, the research reveals how the receptor's activation differs from its human counterpart, highlighting unique evolutionary features. These findings offer valuable insights for developing GABA_B_‐targeted allosteric modulators with therapeutic potential for medication and insecticides.

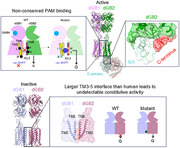

## 1 Introduction

The GABA_B_ receptor is a G protein‐coupled receptor (GPCR) that responds to γ‐aminobutyric acid (GABA), the principal inhibitory neurotransmitter in the central nervous system. GABA_B_ receptor couples to G_i/o_ protein to regulate synaptic transmission.^[^ ^1^ ^]^ Activation of GABA_B_ receptors on presynaptic terminals leads to decreased release of excitatory neurotransmitters through inhibition of calcium (Ca^2+^) channels.^[^ ^2^ ^]^ When the GABA_B_ receptor is activated on postsynaptic sites, it results in the opening of potassium (K^+^) channels, leading to hyperpolarization of the neurons.^[^ ^3^ ^]^ Disruption of GABA_B_ receptor signaling is involved in several neuropsychiatric conditions, such as epilepsy, anxiety, depression, schizophrenia, addiction, and pain.^[^ ^4^ ^]^ Genetic mutations in the GABA_B_ receptor are associated with Rett syndrome, epileptic encephalopathy, and infantile epileptic spasms.^[^ ^5^ ^]^ Baclofen, a selective agonist of the GABA_B_ receptor, is commonly prescribed for managing spasticity in patients with multiple sclerosis and for the treatment of alcohol dependence.^[^ ^6^ ^]^ Previous studies indicate that positive allosteric modulators (PAMs) such as rac‐BHFF, can effectively modulate GABA_B_ receptor function with fewer side effects in vivo.^[^ ^7^ ^]^ Recently, we reported the first negative allosteric modulator (NAM) of the GABA_B_ receptor, CLH304a, which inhibits GABA_B_ receptor activity in neurons.^[^ ^8^ ^]^ However, there are no approved PAM or NAM drugs targeting the GABA_B_ receptor in the market.

GABA_B_ receptor belongs to the class C GPCR, a family of 22 genes in human^[^ ^9^ ^]^ which comprise the metabotropic glutamate receptors (mGluRs), the calcium‐sensing receptor (CaSR), and the sweet and umami taste receptors.^[^ ^10^ ^]^ Most of these receptors form constitutive dimers to be functional. The endogenous ligands are recognized by a large extracellular Venus flytrap (VFT) domain made by two lobes that close upon ligand binding. This domain is connected to a seven‐transmembrane (7TM) domain, which is a characteristic feature of all GPCRs, via an extracellular linker domain. Among the class C GPCRs, GABA_B_ has unique features, forming an obligatory heterodimer composed of two homologous subunits, GB1 and GB2.^[^ ^11^ ^]^ Each subunit consists of VFT and 7TM connected by an extracellular stalk domain. The VFT domain of GB1 is responsible for agonist and antagonist recognition,^[^ ^12^ ^]^ while the 7TM domain in GB2 couples to G_i/o_ protein.^[^ ^13^ ^]^ The binding of GABA to the VFT domain of the GB1 triggers a rearrangement of the inactive 7TM interface through TM3‐5 to the active 7TM interface through TM6, which in turn induces the coupling of G_i_ protein through intracellular loop (ICL) 1–3 and TM3 of GB2.^[^ ^14^ ^]^ PAM (rac‐BHFF) interacts with the interface formed by TM5 and TM6 of GB1 and TM6 of GB2.^[^ ^7^, ^14^ ^]^ The C‐terminal regions of the GABA_B_ receptor are reported to be involved in the receptor trafficking but not receptor signaling.^[^ ^13^, ^15^ ^]^ GB1 contains an R‐x‐R‐type endoplasmic reticulum (ER) retention motif in its cytoplasmic tail,^[^ ^11^, ^16^ ^]^ near the C‐terminal end of a coiled‐coil domain,^[^ ^16^, ^17^ ^]^ The formation of this coiled‐coil domain between GB1 and GB2 masks this retention motif, facilitating receptor expression at the cell surface.^[^ ^18^ ^]^

GABA_B_ receptor is widely distributed across various species including actiniae (sea anemone *Nematostella vectensis)*, sponges (*Leucandra aspera)*, worms (*Caenorhabditis elegans*), lampreys (*Petromyzon marinus*), insects (*Drosophila melanogaster, Periplaneta americana*), fishes (*Danio rerio*), batrachians (*Xenopus tropicalis*), and birds.^[^ ^19^ ^]^ Studies indicate that most of these species possess two homologous genes of the GABA_B_ receptor. In *Caenorhabditis elegans*, we and others show that GBB‐1 (*C. elegans* GB1) alone regulates aging through G_i/o_ protein.^[^ ^19^, ^20^ ^]^ *Drosophila melanogaster* possesses three homologous genes of GABA_B_ receptor, dGB1, dGB2, and dGB3.^[^ ^19a^ ^]^ Whereas *drosophila* GABA_B_ receptor composed of dGB1 and dGB2 regulates circadian rhythm circuits^[^ ^21^ ^]^ and sleep^[^ ^22^ ^]^ through G_i/o_ proteins,^[^ ^19^, ^23^ ^]^ the function of dGB3 remains unknown.^[^ ^22^ ^]^ We have shown that dGB2 alone does not express at the cell surface, and dGB1 can bring dGB2 to the cell surface through the conserved coiled‐coil domain interaction between dGB1 and dGB2, thus revealing a distinct quality control mechanism of GABA_B_ receptor during evolution.^[^ ^24^ ^]^ Whether the activation mechanism of the GABA_B_ receptor is conserved during evolution remains unknown. Comparing their structures from different species may help characterize the receptor activation trajectory and guide future PAM and NAM drug design.

Here, we determined the cryo‐EM structures of the *drosophila* GABA_B_ receptor both in CGP54626 (a competitive antagonist)‐bound inactive state and GABA (agonist)‐bound active state in complex with heterotrimeric G_i_ protein. Although *drosophila* GABA_B_ receptor activity is also controlled by its inactive and active interface of dGB1 and dGB2 and exhibits a similar asymmetric activation of G_i_ protein by dGB2 as done by its human homolog, several important insights have been obtained. A larger inactive interface through TM3‐5 of two subunits is responsible for preventing constitutive activity of the *drosophila* GABA_B_ receptor, revealing the importance of the inactive 7TM interface to control GABA_B_ receptor constitutive activity. Most residues within the binding pocket of PAM in the active interface are conserved between *drosophila* and human GABA_B_ receptor, suggesting the binding mode of PAM is similar. We further identify four non‐conserved residues that are important for PAM activity in the human GABA_B_ receptor. The intracellular loop (ICL) 2 is crucial for class C GPCR G_i_ protein coupling.^[^ ^13^, ^25^ ^]^ The ordered C terminus of dGB2 with ICL1, ICL3 and TM3 form a pocket to permit G_i_ protein coupling, whereas ICL2 of dGB2 is less involved. Interestingly, deletion of the ordered C‐terminus of dGB2 and the corresponding region of its human homolog impairs receptor activation, indicating that ICLs cooperate with the C‐terminus of GB2 to activate the G_i_ protein in both species. In all, our findings provide a more complete understanding of the activation mechanism of GABA_B_ receptor through comparing the evolutionary variations of the structures and new insights for future development of GABA_B_ receptor PAM or NAM for medication and insecticides, by targeting its inactive or active 7TM interface.

## 2 Results

### 2.1 The Overall Architecture of the Drosophila GABA_B_ Receptor is Conserved

**Figure 1:**
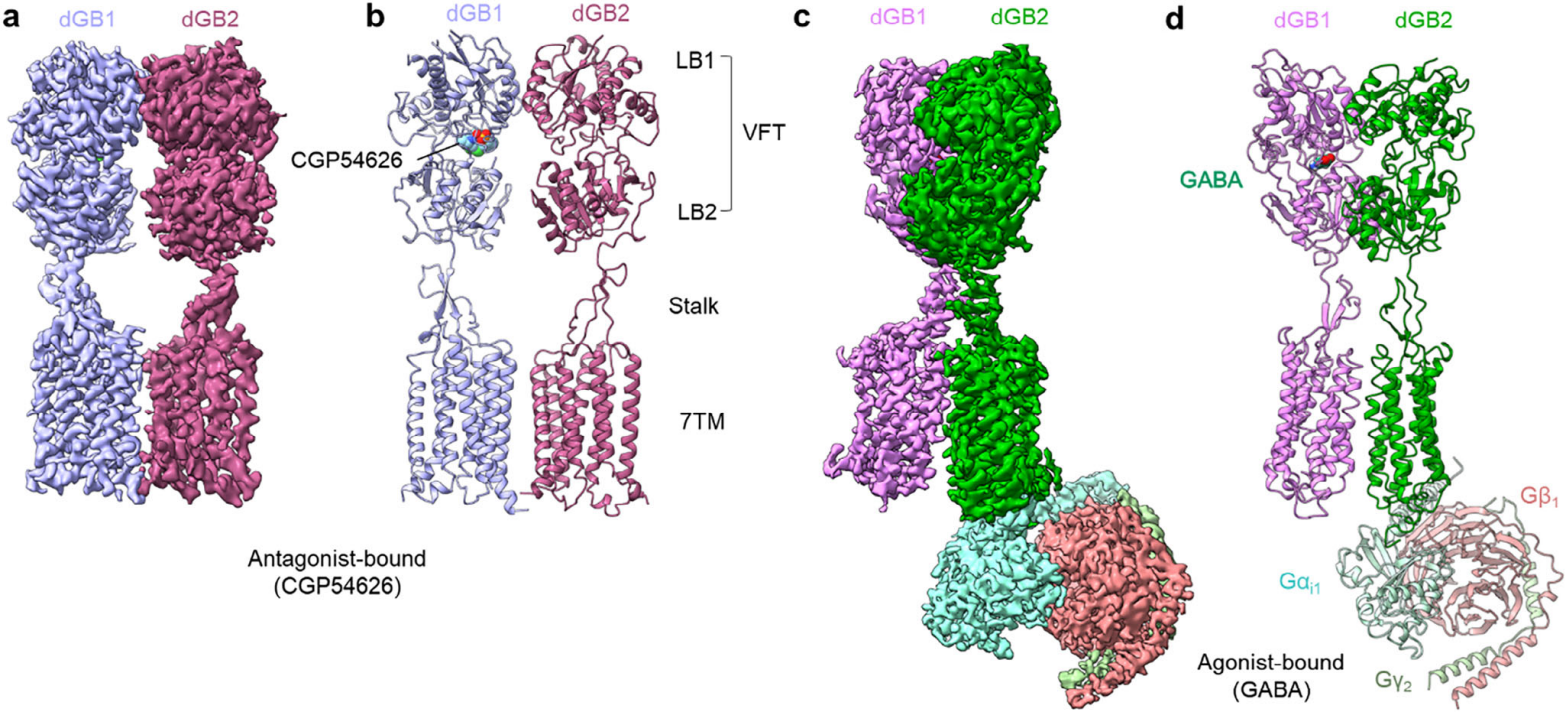
Cryo‐EM maps and models of the *drosophila* GABA_B_ receptor. a,b) Cryo‐EM map (a) and model (b) of the antagonist‐bound *drosophila* GABA_B_ receptor. c,d) Cryo‐EM map (c) and model (d) of the agonist‐bound *drosophila* GABA_B_ receptor in complex with heterotrimeric G_i_ proteins composed of Gα_i1_, Gβ_1_, and Gγ_2_. Both of dGB1 and dGB2 subunits are composed of the extracellular VFT domain (LB1 and LB2), the stalk domain, and the 7TM domain.

We developed a strategy to produce and purify a functional *drosophila* GABA_B_ receptor composed of dGB1 and dGB2 subunits. We referred to previously established methods to obtain cryo‐EM structures of the *drosophila* GABA_B_ receptor.^[^ ^14b,c^ ^]^ dGB1 and dGB2 were expressed and assembled in baculovirus‐infected mammalian cells. The C‐terminal coiled‐coil domain of each subunit was truncated to eliminate flexible regions, denoted as EM constructs (Figure S1a, Supporting Information).^[^ ^24^ ^]^ To verify the correct folding and function of the reconstituted *drosophila* GABA_B_ heterodimer, we evaluated GABA_B_ receptor‐induced G protein activation by measuring the GABA‐induced intracellular Ca^2+^ release in HEK293 cells co‐transfected with dGB1, dGB2, and the chimeric G protein G_qi9_, which facilitates phospholipase C activation.^[^ ^11c^ ^]^ G protein activation of the EM constructs was comparable to that of the wild‐type receptor (Figure S1b, Supporting Information). This indicates that the removed regions in the cytoplasmic tail are not essential for GABA_B_ receptor activation. After detergent extraction, affinity chromatography, and size‐exclusion chromatography, two sets of high‐purity samples were obtained and verified by SDS‐PAGE and negative staining (Figure S1d–i, Supporting Information). Data collection using cryo‐EM was conducted, followed by the analysis, processing, and modeling of the acquired data (**Table** **1**, Figure S2a–d, Supporting Information), resulting in the successful generation of cryo‐EM structural models of the *drosophila* GABA_B_ receptor: a 3.52 Å model of CGP54626‐bound form (**Figure** **1**a,b; Figure S3a,b, Supporting Information) and a 3.3 Å model of GABA‐bound form in complex with the heterotrimeric G_i1_ protein (Figure 1c,d; Figure S3c,d, Supporting Information).

**Table 1:** Cryo‐EM data collection, refinement, and validation statistics.

	Inactive DGABAB (EMDB‐61742) (PDB 9JQX)	DGABAB‐Gi (EMDB‐61745) (PDB 9JR0)
Data collection and processing		
Magnification	130 000×	130 000×
Voltage [kV]	300	300
Electron exposure [e^−^ Å^−2^)	50	50
Defocus range [µm]	−1–−2.5	−1–−2.5
Pixel size [Å]	0.93	0.93
Symmetry imposed	C1	C1
Initial particle images [no.]	4 664 233	3 023 662
Final particle images [no.]	36 119	299 674
Map resolution [Å] FSC threshold	3.52 0.143	3.3 0.143
Map resolution range [Å]	2–4	2–4
Refinement		
Initial model used (PDB code)	7C7S	7EB2
Model resolution [Å] FSC threshold	3.52 0.5	3.3 0.5
Model resolution range [Å]	2.7–3.8	2.8–3.4
Model composition Non‐hydrogen atoms Protein residues Ligands	10 765 1356 1	14 679 2030 1
*B* factors [Å^2^] Protein Ligand	45.36 28.35	65.06 7.02
R.m.s. deviations Bond lengths [Å] Bond angles [°]	0.007 0.964	0.003 0.57
Validation MolProbity score Clashscore Poor rotamers [%]	2.03 12 0	1.79 8.57 0.15
Ramachandran plot Favored [%] Allowed [%] Disallowed (%)	93.24 6.76 0	95.34 4.66 0

The overall architecture of the *drosophila* GABA_B_ receptor revealed a heterodimeric arrangement of dGB1 and dGB2 (Figure 1a–d), with each subunit consisting of an extracellular VFT connected to the 7TM domain via the stalk domain. Each extracellular and intracellular loop (ECL and ICL, respectively) interconnects adjacent helices within the TM domains and is visible. The cytoplasmic C‐termini of both dGB1 and dGB2 are partially visible in the density map, excluding the coiled‐coil domain. The structures of *drosophila* GABA_B_ VFT domains in CGP54626‐bound form and in GABA‐bound form are highly similar to those of the human GABA_B_ structures in inactive (PDB 7C7S) and active states (PDB 4MS3),^[^ ^12^, ^14^ ^]^ as previously reported, with root mean squared deviation (RMSD) of 1.136 (between 366 pruned atom pairs) and 0.892 Å (between 351 pruned atom pairs), respectively (Figure S4a,b, Supporting Information). Therefore, we presume that our CGP54626‐bound *drosophila* GABA_B_ receptor structure represents the inactive state, whereas the GABA‐bound *drosophila* GABA_B_ receptor structure in complex with G_i1_ proteins represents the active state.

The VFTs of both dGB1 and dGB2 consist of two lobes (LB1 and LB2). In the inactive state, the lobes of the dGB1 VFT are open, whereas in the active state, they close. In contrast, the lobes of the dGB2 VFT remain open in both the inactive and active states (Figure 1b,d). Activation of the *drosophila* GABA_B_ receptor induces large conformational changes in the dGB1 and dGB2 heterodimer. This includes the rearrangement of the LB2 in the VFT, which shortens the distance between the C‐termini of dGB1 VFT (residue D453) and dGB2 VFT (residue D432) from 46 to 31.5 Å (Figure S5a, Supporting Information). This resulted in the rearrangement of the 7TM interface, changing from TM3‐5/TM3‐5 to TM6/TM6 via movement of the stalk domains (Figure S5a,b, Supporting Information).

*Drosophila* GABA_B_ receptor has a conserved ligand‐binding pocket within the VFT domain of dGB1. The residues within the ligand‐binding pocket that interact with CGP54626 were W51, S116, S139, H156, and E343 in LB1 and W264 in LB2, whereas the corresponding residues in human GB1 were W182, S247, S270, H287, and E466 in LB1 and W395 in LB2 (Figure S4a, Supporting Information). The residues within the pocket that interacted with GABA were W51, S116, S139, H156, and E343 in LB1, and Y236 and W264 in LB2, whereas the corresponding residues in human GB1 were W65, S130, S153, H170, and E349 in LB1, and Y250 and W278 in LB2 (Figure S4b, Supporting Information). We verified whether conservation of the ligand‐binding pocket agreed with the pharmacology of the *drosophila* GABA_B_ receptor. Our results show that similar GABA‐induced activation can be inhibited by CGP54626, but the inhibitory efficacy of CGP54626 on the *drosophila* GABA_B_ receptor is weaker than that on the human GABA_B_ receptor, with a 46.3‐fold increase in the half‐maximal inhibitory concentration (IC_50_) (Figure S4c,d, Table S1, Supporting Information), consistent with the previous report.^[^ ^19a^ ^]^

Although residues within the ligand‐binding pocket of dGB1, as well as the surrounding residues, are nearly identical to those of human GB1, the differential efficacy of CGP54626 remains unexplained. This discrepancy may result from additional residues that do not directly contact with CGP54626 but influence its binding indirectly. Another possible explanation is that the GABA_B_ receptors in mammals and *drosophila* function as distinct molecular machines, with differences in their conformational dynamics or in the coupling efficiency to downstream signaling components, leading to altered pharmacological profiles.

### 2.2 Larger Inactive 7TM Interface is Responsible for the Undetectable Constitutive Activity in *Drosophila* GABA_B_ Receptor

**Figure 2:**
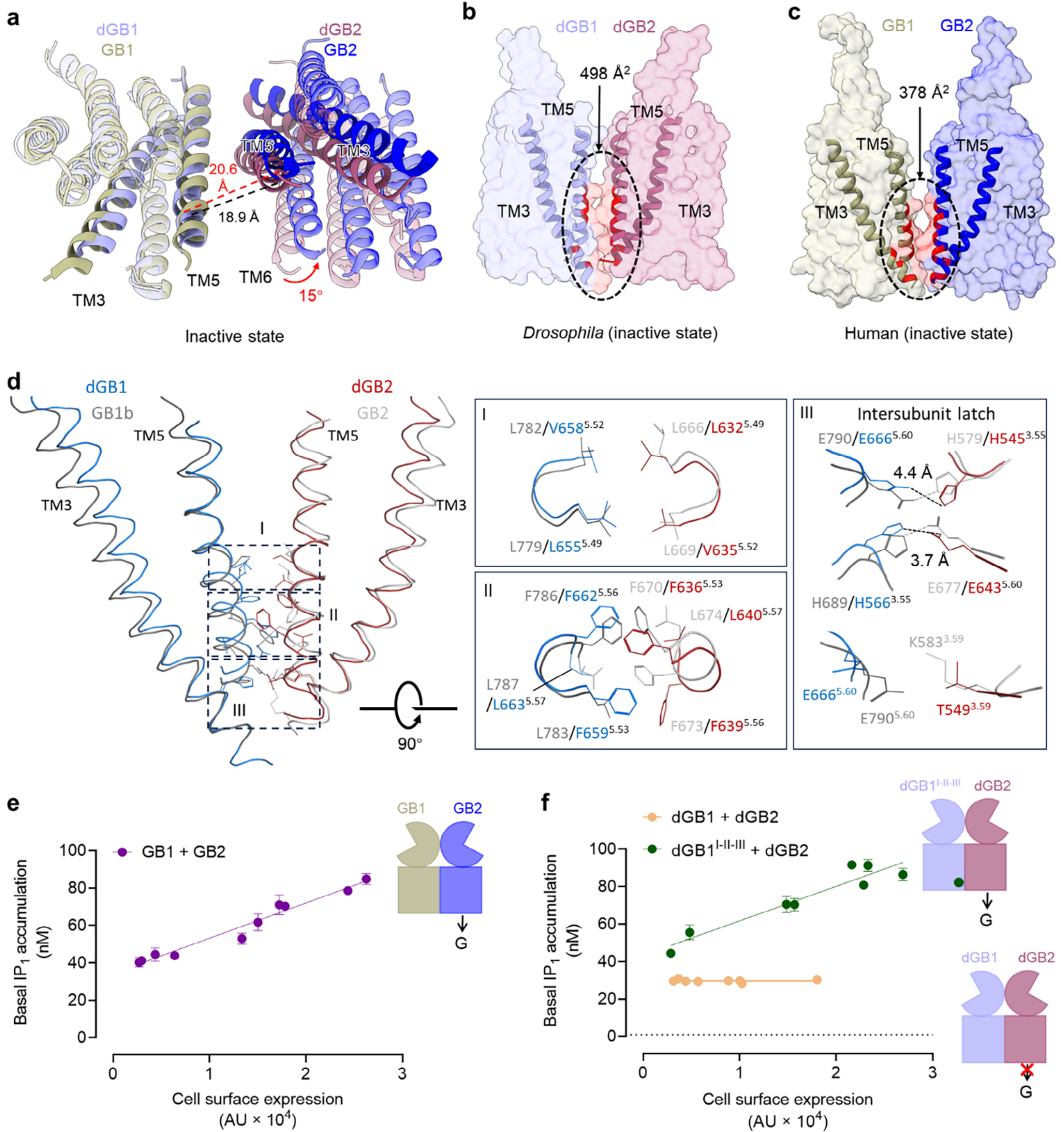
Structural insights into the constitutive activity of GABA_B_ receptor. a) Overlay of the structures of *drosophila* and human GABA_B_ receptors 7TM domains in the inactive state. Dashed lines indicate the distances between the TM5 regions of the 7TM interface based on the C_α_ atoms of dGB1‐L646^5.40^ and dGB2‐V623^5.40^ (black dotted line), as well as GB1‐L770^5.40^ and GB2‐L657^5.40^ (red dotted line). b,c) Surface representations of 7TM domains of *drosophila* and human GABA_B_ receptors in the inactive state. The interaction areas between 7TMs were highlighted in dotted circles. The interaction areas were calculated and labeled. d) The structural comparison of the inter‐subunit contacts within the TM3‐5 interface of *drosophila* and human GABA_B_ receptors in the inactive state. Three layers (I–III) of inter‐subunit contacts are highlighted within dotted boxes and are enlarged on the right. Residues in the layers are labeled. The potential salt bridges forming the inter‐subunit latch are highlighted in dotted lines. e,f) Basal IP_1_ accumulation of human GABA_B_ receptor heterodimer (e), or *drosophila* GABA_B_ receptor mutant (dGB1^I‐II‐III^ + dGB2) but not to the wild‐type (dGB1 + dGB2) (f). Data are representative of one experiment performed in triplicate and repeated independently three times with similar results; data are present as mean ± S.E.M.

In the human GABA_B_ receptor, a rearrangement of the 7TM interface is crucial for receptor activation.^[^ ^14a^ ^]^ The 7TM interfaces of *drosophila* GABA_B_ receptor in the inactive and active states are formed through TM3‐5 and TM6, respectively (Figure S5a,b, Supporting Information), indicating that the activation of *drosophila* GABA_B_ receptor induces a similar rearrangement of the 7TM interface from TM3‐5 to TM6, similar to its human homolog (Figure S5c,d, Supporting Information). However, the stalk domain of dGB2 in the inactive *drosophila* GABA_B_ receptor is displaced by ≈7.5 Å relative to GB2 of the inactive human GABA_B_ receptor (Figure S6a,b, Supporting Information). This displacement results in a relative shift of the TM interface in the *drosophila* GABA_B_ receptor compared with its human homolog, which includes a 15° rotation of 7TM (**Figure** **2**a; Figures S5c,d,S6c, Supporting Information) and a reduction in the distance between TM5/TM5, changing from 20.6 to 18.9 Å (Figure 2a). We propose that these relative shifts increase the 7TM interface area in the inactive *drosophila* GABA_B_ receptor, which is ≈32% larger than that in the inactive human GABA_B_ receptor (PDB 7C7S) (498 Å^2^ vs 378 Å^2^) (Figure 2b,c). In the active state, the stalk domain of the dGB2 is shifted by ≈2.1 Å relative to the active human GB2 (Figure S6d,e, Supporting Information), leading to a 5.2 Å shift of 7TM in the active dGB2 compared with the active human GB2 (Figure S6f, Supporting Information). These relative shifts did not alter the 7TM interface area of the active *drosophila* GABA_B_ receptor. The interaction areas of the 7TM interface of GABA‐bound *drosophila* and agonist/PAM‐bound human GABA_B_ receptors were similar (PDB 7EB2) (722 and 704 Å^2^, respectively) (Figure S5e–g, Supporting Information).^[^ ^14c^ ^]^

In the CGP54626‐bound inactive state, *drosophila* GABA_B_ receptors exhibit three distinct inter‐subunit interface layers, consistent with the human homolog (Figure 2d).^[^ ^14^, ^26^ ^]^ Layer I comprises hydrophobic interactions involving leucines and valines (L655^5.49^ and V658^5.52^ in dGB1; L632^5.49^ and V635^5.52^ in dGB2). Although these residues are not fully conserved – human GB1 and GB2 subunits feature four leucines – their hydrophobic interactions remain comparable. Layer II involves the tight packing of phenylalanine and leucine residues, including F659^5.53^, F662^5.56^, and L663^5.57^ in dGB1 (L666^5.53^, F669^5.56^, and L670^5.57^ in GB1), and F636^5.53^, F639^5.56^, and L640^5.57^ in dGB2 (F670^5.53^, F673^5.56^, and L674^5.57^ in GB2). A key difference lies at position 5.53, where leucine in the human receptor is substituted with phenylalanine in *drosophila*, introducing a bulkier side chain that increases contact surface area and may enhance aromatic stacking interactions. Layer III, located at the intracellular end of the interface, consists of a network of salt bridges, including dGB1‐H566^3.55^/dGB2‐E643^5.60^ and dGB1‐E666^5.60^/dGB2‐H545^3.55^, which function as the inter‐subunit latch. Additionally, a potential polar interaction is also observed in *drosophila* receptors between dGB1‐E666^5.60^ and dGB2‐T549^3.59^ (equivalent to hGB2‐K583^3.59^), further suggesting that layer III is highly conserved in both human and *drosophila* GABA_B_ receptors.

Taken together, our findings demonstrate that the *drosophila* GABA_B_ receptor retains inter‐subunit interaction features similar to those of the human receptor at the inactive TM3–5 interface. However, distinct conformational shifts and amino acid substitutions, particularly within the 7TM region, result in a markedly larger interface area in the inactive *drosophila* receptor. This expanded interface likely stabilizes the receptor in an inactive conformation, thereby preventing constitutive activation. Supporting this, basal IP_1_ accumulation was proportional to the amount of human GABA_B_ receptor expressed on the cell surface (Figure 2e), whereas no such accumulation was detected for the *drosophila* receptor (Figure 2f), indicating undetectable constitutive activity. These observations are consistent with previous structural and mutational studies of the human receptor, highligting the critical role of these inter‐subunit interactions in maintaining receptor inactivity.

To verify whether the larger inactive 7TM interface was responsible for the undetectable constitutive activity, we generated a dGB1 double mutant within Layer III to disrupt the inter‐subunit latch (E666A and H566A, denoted as dGB1^III^). Although the cell surface expression of dGB1^III^ when transfected together with wild‐type dGB2 in HEK293 cells was lower than that of the wild‐type *drosophila* GABA_B_ receptor (Figure S5h,i, Table S2, Supporting Information), dGB1^III^ restored the constitutive activity of *the drosophila* GABA_B_ receptor (59% increase in IP_1_ accumulation compared with wild‐type, *P* = 0.0022) (Figure S5j, Table S2, Supporting Information), demonstrating that the inter‐subunit latch is important for controlling receptor constitutive activity as in its human homolog.^[^ ^14^, ^26^ ^]^ We further generated other dGB1 mutants within Layers I and II of the inactive 7TM interface by introducing a non‐conserved residue from human GB1 into the corresponding sites of dGB1 (V658L in Layer I, denoted as dGB1^I^; F659L in Layer II, denoted as dGB1^II^). The cell surface expression levels of these mutants transfected with wild‐type dGB2 were similar to those of the wild‐type receptor (Figure S5h,i, Table S2, Supporting Information). Our data showed that dGB1^I^ and dGB1^II^ were also able to restore the constitutive activity of *the drosophila* GABA_B_ receptor (50% and 66% increase in IP_1_ accumulation compared with the wild‐type receptor, *P* = 0.0085 and *P* = 0.0008, respectively) (Figure S5j, Table S2, Supporting Information), demonstrating that Layers I and II are also important for controlling *drosophila* GABA_B_ receptor constitutive activity. Finally, we generated two sets of mutants: dGB1^I‐II^ with V658L in Layer I and F659L in Layer II, and dGB1^I‐II‐III^ with V658L in Layer I, F659L in Layer II, and E666A and H566A in Layer III. The cell surface expression levels of dGB1^I‐II^, when transfected with wild‐type dGB2, were similar to those of the wild‐type receptor, whereas the cell surface expression of dGB1^I‐II‐III^ when transfected with wild‐type dGB2 in HEK293 cells was lower than that of the wild‐type receptor (Figure S5h,i, Table S2, Supporting Information). Both dGB1^I‐II^ and dGB1^I‐II‐III^ resulted in higher constitutive activity than dGB1^III^ (17% and 22% increase in IP_1_ accumulation compared with dGB1^III^, *P* = 0.0249 and *P *= 0.0044, respectively) (Figure S5j, Table S2, Supporting Information), further confirming the role of inter‐subunit interactions in Layers I and II in controlling constitutive activity. Interestingly, the IP_1_ accumulation was also proportional to the level of receptor expression on the cell surface of HEK293 cells co‐expressing dGB1^I‐II‐III^ and wild‐type dGB2 (Figure 2f). Overall, we propose that a larger interface area composed of Layers I, II, and III in TM3‐5 prevents the constitutive activity of *the drosophila* GABA_B_ receptor.

### 2.3 The key Residues within the Active 7TM Interface for PAM Activity are not Conserved in the *Drosophila* GABA_B_ Receptor

**Figure 3:**
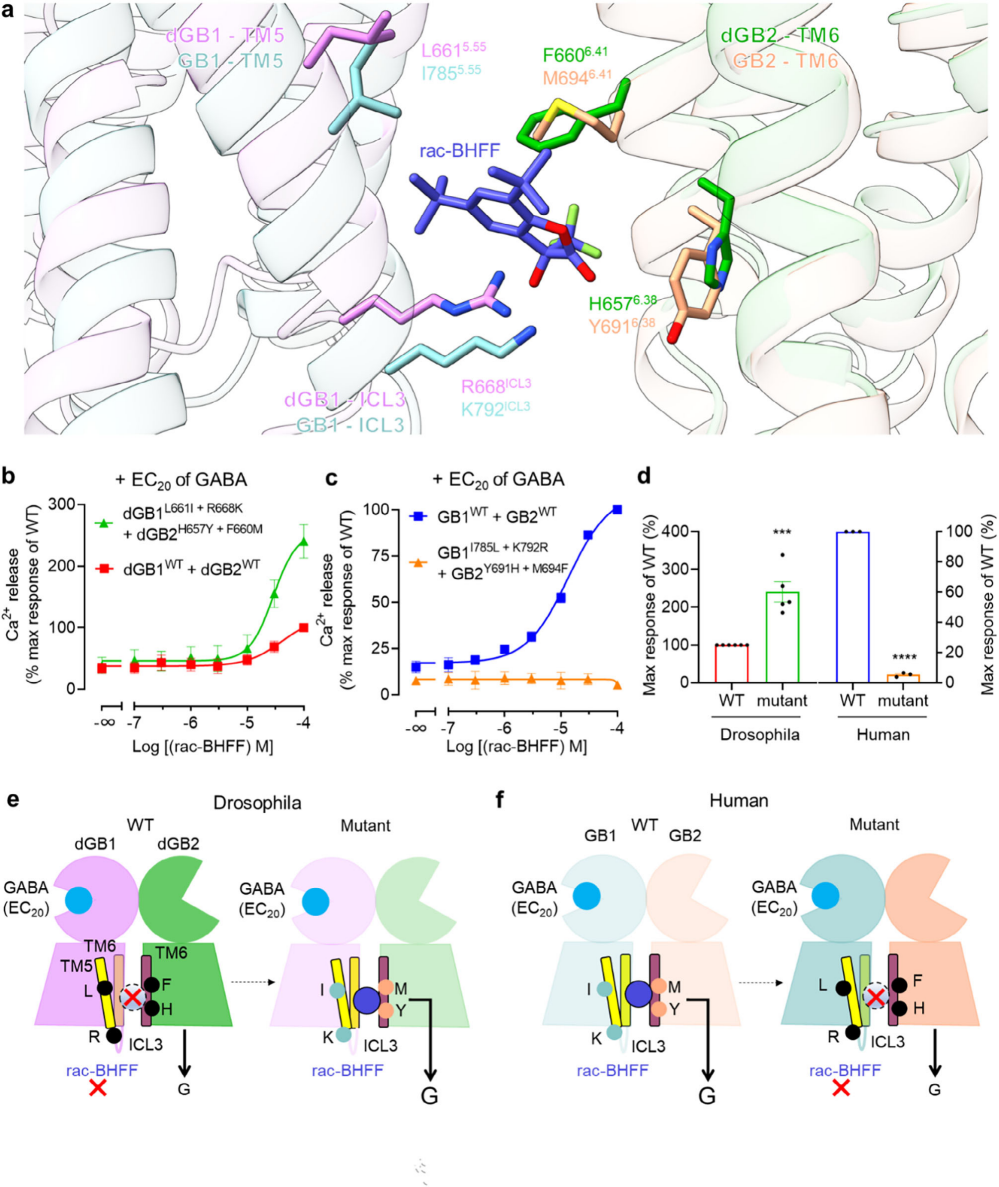
The allosteric agonism by PAM is conserved in the drosophila GABA_B_ receptor. a) Side view of the superimposed structures of the agonist and PAM‐bound human GABA_B_ receptor (PDB 7EB2) and GABA‐bound drosophila GABA_B_ receptor, aligned by the 7TM domain of the GB2 subunit. b,c) Intracellular calcium release induced by rac‐BHFF in HEK293 cells co‐transfected with the indicated constructs in the presence of EC_20_ of GABA. Data are normalized by the wild‐type response. d) Bars represent differences in calculated maximal efficacy (*E*_max_) for drosophila and human GABA_B_ receptors as a percentage of the maximum response. Data in (b–d) are shown as means ± SEM of at least three biologically independent experiments, performed in technical triplicate, and analyzed using an unpaired *t‐*test (two‐tailed). e,f) Schematic representations of the allosteric agonism by PAM in drosophila (e) and human (f) GABA_B_ receptors. The four non‐conserved residues in TM5, ICL3, and TM6 were highlighted and labeled.

In the human GABA_B_ receptor, it was demonstrated that PAM (rac‐BHFF) binds to the active 7TM interface.^[^ ^14c^ ^]^ Residues located in TM5 (I785, A788, and Y789), ICL3 (K792) and TM6 (R803, G806, M807, and Y810) of GB1, as well as TM6 (K690, Y691, M694, and N698) and TM7 (L738) of GB2, form the rac‐BHFF‐binding pocket (Figure S7a, Supporting Information). Amino acid sequence alignment and superimposition of the structures of active human and *drosophila* GABA_B_ receptors showed that most of the residues located within the rac‐BHFF binding pocket in the human GABA_B_ receptor were conserved in the *drosophila* GABA_B_ receptor (**Figure** **3**a; Figure S7a, Supporting Information). These non‐conserved residues include I785^5.55^ and K792^ICL3^ of GB1, as well as Y691^6.38^ and M694^6.41^ of GB2 in the human GABA_B_ receptor (the corresponding residues in the *drosophila* GABA_B_ receptor are L661^5.55^ and R668^ICL3^ of dGB1, and H657^6.38^ and F660^6.41^ of dGB2).

To investigate the conservation of PAM effects on GABA_B_ receptor activity, we measured the intracellular Ca^2+^ release induced by rac‐BHFF in the presence of EC_20_ GABA in HEK293 cells co‐transfected with dGB1, dGB2, and the chimeric G protein G_qi9_. Our data showed that rac‐BHFF had no effect on the *drosophila* GABA_B_ receptor (Figure 3b,d, Table S3, Supporting Information). To verify whether these four non‐conserved residues are the key residues responsible for rac‐BHFF activity, we generated two sets of mutants to analyze the effect of PAM on GABA_B_ receptor activity during evolution. The *drosophila* GABA_B_ receptor mutants were generated by introducing four non‐conserved residues from the human homolog into the corresponding sites of *the drosophila* GABA_B_ receptor (L661I^5.55^‐R668K^ICL3^ in dGB1 and H657Y^6.38^‐F660M^6.41^ in dGB2). Human GABA_B_ receptor mutants were generated by replacing the four non‐conserved residues with the corresponding residues from the *drosophila* GABA_B_ receptor (I785L^5.55^‐K792R^ICL3^ of GB1 and Y691H^6.38^‐M694F^6.41^ of GB2). Interestingly, rac‐BHFF showed a strong PAM effect in the activities of *drosophila* GABA_B_ receptor mutants (Figure 3b,d; Figure S7b–e, Table S3, Supporting Information), whereas the PAM effect of rac‐BHFF disappeared in the human GABA_B_ receptor mutants (Figure 3c,d; Figure S7f–i, Table S3, Supporting Information). These data demonstrate that I785^5.55^ and K792^ICL3^ in GB1, as well as Y691^6.38^ and M694^6.41^ in GB2, are the key residues that confer PAM effects on GABA_B_ receptor activity (Figure 3e,f).

### 2.4 The ICL2 of *Drosophila* GB2 is Less Involved in G Protein Coupling

**Figure 4:**
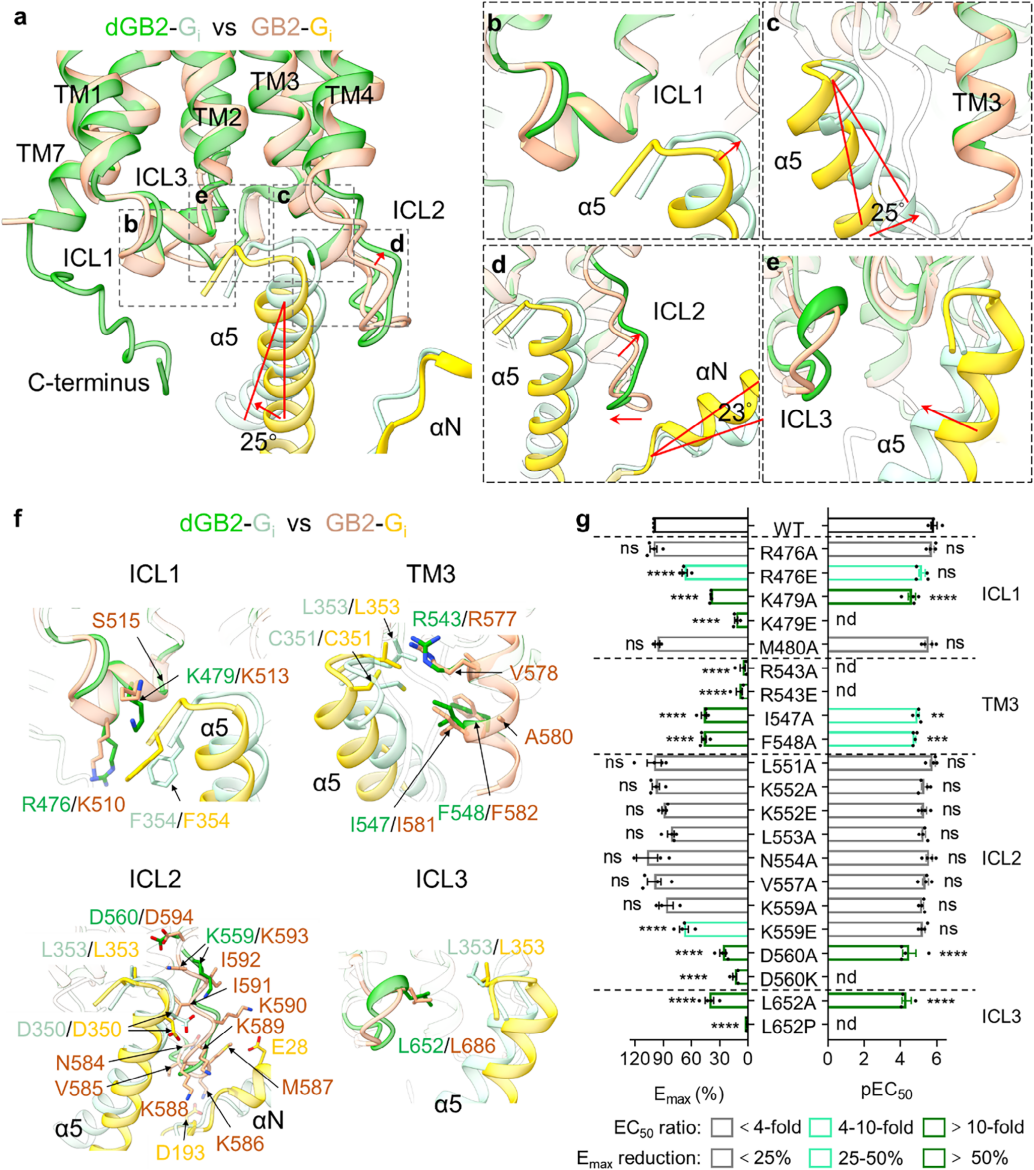
The G protein binding pocket of the *drosophila* GABA_B_ receptor. a) Overview of the G_i1_ protein‐binding pockets of *drosophila* and human GABA_B_ receptor. b–e) The enlarged structural comparisons of the interactions between the ICL1 (b), TM3 (c), ICL2 (d), and ICL3 (e) of dGB2 (green) or GB2 (brown), and the G_i1_ protein. f) Structural insights into the G_il_ protein‐binding pocket of *drosophila* and human GABA_B_ receptor. Residues involved in the G protein coupling are indicated. g) Intracellular calcium release by the wild‐type and mutant dGB2 coexpressed with the wild‐type dGB1 and the chimeric G protein Gqi9 in the presence of various concentrations of GABA. Bars represent differences in calculated maximal efficacy (*E*_max_) and potency (pEC_50_) for each mutant as a percentage of the maximum in wild‐type *drosophila* GABA_B_ receptor. Data are normalized by wild‐type response. Values are shown as means ± SEM of at least three biologically independent experiments, performed in technical triplicate, and analyzed using one‐way analysis of variance with Dunnett's multiple comparison test to determine significance (compared with wild‐type). Nd, not determined. Ns, not significant.

The overall structure of the active *drosophila* GABA_B_‐G_i1_ complex exhibited high similarity to that of the active human GABA_B_‐G_i1_ complex (PDB 7EB2), with an RMSD of 1.24 Å (between 463 pruned atom pairs) (Figure S6d, Supporting Information). Whereas the α5 helix of G_i1_ is stabilized within a pocket formed by TM3, ICL1, ICL2, and ICL3 in its human homolog, the resolved ordered C‐terminus of dGB2 also participates in the pocket formation (**Figure** **4**a). Amino acid sequence alignment showed that the residues in the G_i1_ protein‐binding pocket of human GB2 were highly conserved in dGB2 (Figure S8a,b, Supporting Information). However, the number of residues at the interaction interface between G_i1_ and dGB2 was lower than that observed with GB2. These include residues S515 in ICL1, V578, and A580 in TM3 of GB2, which are present at the G‐protein coupling interface, whereas the corresponding residues S481, V544, and S546 in dGB2 are absent from this interface. Additionally, residues N584, K589, K590, and I592 in GB2 were present at the G protein coupling interface, whereas the corresponding residues D550, K555, K556, and I558 in ICL2 of dGB2 were absent from this interface (Figure S8a, Supporting Information). In comparison with the structure of human GABA_B_‐G_i1_ complex, differences are observed in the TM3, ICL1, ICL2, and ICL3 regions of dGB2, as well as in the α5 and αN helices of *drosophila* GABA_B_‐G_i1_ complex (Figure 4a–e). The ICL1 in dGB2 is primarily in contact with the C‐terminal hooked region of the α5 helix of G_i_, with the difference that α5 helix of G_i_ in *drosophila* GABA_B_‐G_i1_ complex is rotated by 25° (Figure 4a,c), resulting in the top of the C‐terminal hooked region being further away from ICL1 (Figure 4b). The TM3 of human GB2 and dGB2 are well matched, and the deflected‐ α5 helix is close to the TM3 (Figure 4c). Although the deflected α5 is relatively close to the ICL3 of dGB2 (Figure 4e), it is still located far away and binds to ICL3 mainly through the C‐terminal hook region of the α5 helix of G_i_. ICL2 is a major determinant of G protein coupling in class C GPCRs.^[^ ^13^, ^27^ ^]^ In comparison with the structure of human GABA_B_‐G_i1_ complex, the αN helix of the G_i1_ protein in the *drosophila* GABA_B_‐G_i1_ complex was deflected downward by 23°, together with a change in the orientation of the tip of ICL2 (Figure 4d), which may result in the fewer interactions between ICL2 of dGB2 and αN helix of G_i1_ protein compared with that of human GB2. Notably, the ICL2 of dGB2 in the inactive state was poorly densified and difficult to resolve.

The detailed interactions between human and *drosophila* GB2 and G_i1_ are consistent with our overall observations. Because the position of the C‐terminal hook‐like region in α5 helix is deflected, residues R476 and K479 in ICL1 of dGB2 interact with F354 in α5 of G_i1_ protein, while residues K510 and K513 in human GB2 interact with F354 in α5 of G_i1_ protein (Figure 4f). Residues R543 in TM3 of dGB2 interact with L353 in α5 of G_i1_ protein, while I547 and F548 forming a hydrophobic cleft, stabilize C351 in α5 of G_i1_ protein, similar to that of the correspondent residues R577, I581, and F582 in human GB2 (Figure 4f). Since ICL2 of dGB2 is positioned away from α5 of G_i1_ protein, only the residue K559 in ICL2 forms a salt bridge with D350 in α5, while D560 in ICL2 interacts with L353 in the C‐terminal hook‐like region of α5 (Figure 4f). In contrast, almost the whole ICL2 region of human GB2 is involved in G‐protein coupling, with key interactions involving two potential salt bridges between lysines in the ICL2 (K586, K589, and K590), and acidic residues in αN (E28) and the linker region in β2–β3 (D193) in G_i1_ (Figure 4f). Additionally, the proximity of ICL3 and α5 does not bring more amino acid residues involved in the interaction, only the conserved L652 in ICL3 of dGB2 (L686 in ICL3 of GB2) interacts with L353 in α5 of G_i1_ protein to stabilize the coupling conformation (Figure 4f).

To evaluate the contribution of these residues in the G protein binding pocket of the *drosophila* GABA_B_ receptor, we substituted these residues in dGB2 to modify their polarity or charge and measured the intracellular Ca^2+^ release induced by GABA in HEK293 cells co‐transfected with wild‐type dGB1, dGB2 mutants, and the chimeric G‐protein G_qi9_. None of these mutations in the G‐protein‐binding pocket of dGB2 attenuated cell surface expression, as detected by ELISA (Figure S8c, Table S4, Supporting Information). The ability of mutants R476E, K479A, and K479E in the ICL1 of dGB2 to activate the G protein was significantly decreased, whereas that of mutant M480A in the ICL1 of dGB2 was not affected (Figure 4g; Figure S8d, Table S4, Supporting Information), suggesting that residues R476 and K479, but not M480 in dGB2, are involved in G‐protein coupling. Mutants R543A, R543E, I547A, and F548A in TM3 largely impaired G‐protein activation (Figure 4g; Figure S8e, Table S4, Supporting Information), indicating that residues R543, I547, and F548 in dGB2 are involved in G‐protein coupling. Only the ability of three of the ten mutants, K559E, D560A, and D560K in ICL2 of dGB2 to activate the G protein was decreased (Figure 4g; Figure S8f, Table S4, Supporting Information), showing that the residues K559 and D560 in dGB2 are involved in G‐protein coupling, similar to the corresponding residues K593 and D594 in human GB2 in our previous analysis.^[^ ^14c^ ^]^ However, the ability of the other seven of the ten mutants in ICL2 of dGB2 to activate the G protein was not affected. Our data indicate that most residues in ICL2 of dGB2 are not involved in G protein coupling, with only 2 of 11 residues impairing this coupling. This is substantially different from the ICL2 regions of human GB2, where 10 out of 11 residues significantly impair G protein coupling when mutated (Figure 4f; Figure S8a, Table S4, Supporting Information). Additionally, mutants L652A and L652P in the ICL3 of dGB2 severely attenuated G protein coupling (Figure 4g; Figure S8g, Table S4, Supporting Information), confirming that residue L652 is involved in G‐protein coupling, similar to the corresponding residue L686 in human GB2 in our previous analysis.^[^ ^14c^ ^]^ In summary, although the G protein coupling pockets in *drosophila* and human GABA_B_ receptors exhibit similarities to stably bind to the partially embedded α5 helix of the G_i1_ protein, ICL2 is less involved in the *drosophila* GABA_B_ receptor G protein coupling.

### 2.5 The Ordered C‐Terminus is Important for G Protein Activation during Evolution

**Figure 5:**
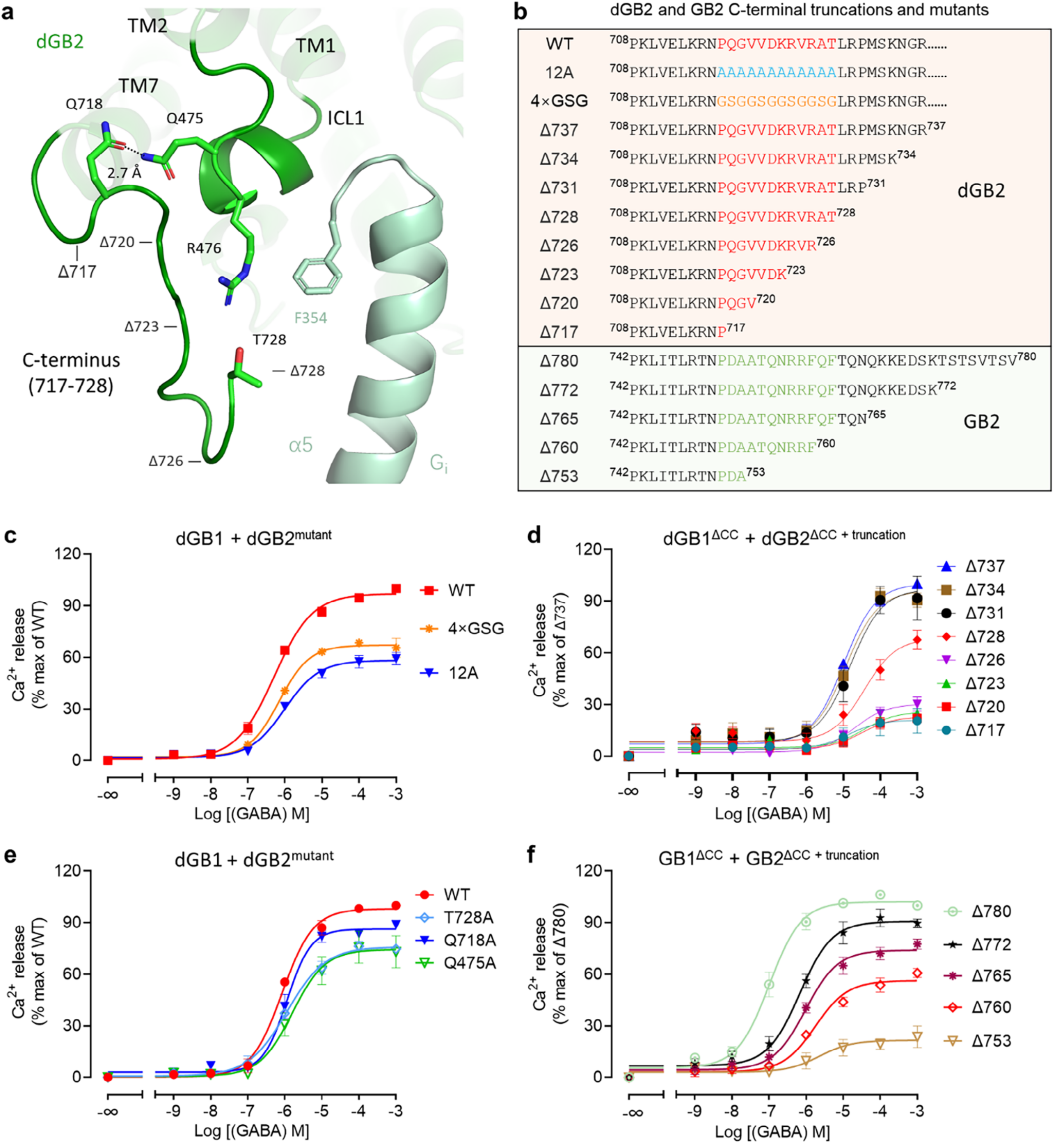
The ordered C‐terminus is involved in G protein coupling. a) The overview of the interaction among the ICL1 and the ordered C‐terminus of the dGB2 subunit (green) and the α5 helix of G_i1_ protein (light green) in the GABA‐bound *drosophila* GABA_B_ receptor–G_i1_ complex. The hydrogen bonds between Q718 and Q475 were indicated in dotted lines. Truncation sites within the ordered C‐terminus of dGB2 were indicated. b) The amino acid sequences of dGB2 and GB2 C‐terminal truncations and mutants. c,e) Intracellular calcium release induced by GABA in HEK293 cells co‐transfected with wild‐type dGB1 and dGB2 and mutants. Data are normalized by the wild‐type response. d,f) Intracellular calcium release induced by GABA in HEK293 cells co‐transfected with dGB1 lacking the coiled‐coil domain (dGB1^ΔCC^) and dGB2 truncations (d), human GB1 lacking the coiled‐coil domain (GB1^ΔCC^) and GB2 truncations (f). Data are normalized by the Δ737 (d) or Δ780 (f) response. Values in (c–f) are shown as means ± SEM of at least three biologically independent experiments performed in technical duplicates.

The C‐termini of GPCRs modulate G protein coupling.^[^ ^28^ ^]^ Although the C‐terminus of GB2 is not observed in the structure of the GABA‐bound human GABA_B_ receptor in complex with G_i1_ protein,^[^ ^14b,c^ ^]^ we identified a resolved ordered C‐terminus of dGB2 located before the coiled‐coil domain and composed of 12 amino acids (residues 717–728) in GABA‐bound receptors in complex with G_i1_ proteins (**Figure** **5**a). To evaluate whether the ordered C‐terminus was involved in G protein coupling, we first replaced these residues with 12 consecutive alanine residues (denoted as 12A), which altered their side‐chain features, or with a combination of four consecutive flexible amino acids, glycine‐serine‐glycine (denoted as 4 × GSG), to modify the secondary structures of the C‐terminus (Figure 5b). Using ELISA, we verified that mutants 12A and 4 × GSG were expressed at equivalent levels on the surface of HEK293 cells compared with the wild‐type (Figure S9a, Table S5, Supporting Information), and then assayed their ability to activate G protein by measuring the intracellular Ca^2+^ release induced by GABA in HEK293 cells co‐transfected with wild‐type dGB1, dGB2 mutants, and the chimeric G protein G_qi9_. We observed that G protein activation by mutants 12A and 4 × GSG was significantly reduced, leading to a substantial reduction of 41% and 30% in maximal responses (*E*_max_) (*P* < 0.0001 and *P *= 0.0001), respectively. Mutant 12A resulted in a twofold reduction in the half‐maximal effective concentration (EC_50_) (*P* = 0.0059), whereas the EC_50_ of mutant 4 × GSG remained unchanged (Figure 5c; Figure S9b, Table S5, Supporting Information). We also evaluated their G protein coupling using bioluminescence resonance energy transfer (BRET) assays based on the dissociation of Gα_i1_ and Gβγ proteins in HEK293 cells, which led to an increase in the BRET ratio.^[^ ^29^ ^]^ We confirmed that both 12A and 4 × GSG mutants of dGB2 significantly reduced GABA‐induced G protein dissociation (12% and 17% reductions in *E*_max_, *P* = 0.0002 and *P* < 0.0001, respectively; 8.8‐fold and threefold reductions in EC_50_, *P* < 0.0001 and *P* = 0.0034, respectively) (Figure S9c, Table S6, Supporting Information). Taken together, our data indicate that the amino acid side chains and the secondary structure of the ordered C‐terminus are important for *drosophila* GABA_B_ receptor coupling to G proteins.

To further assess the contribution of the ordered C‐terminus to G protein coupling, we further generated dGB2 truncated mutants after (Δ737, Δ734, Δ731, and Δ728) and within (Δ726, Δ723, Δ720, and Δ717) the C‐terminus (Figure 5a,b; Figure S9d, Supporting Information). To do so, we measured the intracellular Ca^2+^ release induced by GABA in HEK293 cells co‐transfected with dGB1 mutant lacking the coiled‐coil domain (dGB1^ΔCC^) and various dGB2 truncated mutants. We observed that those truncated mutants of dGB2 within C‐terminus (Δ726, Δ723, Δ720, and Δ717) significantly reduced G protein activation (70–80% reduction in *E*_max_) (Figure 5d; Figure S9e,f, Table S5, Supporting Information) compared with that of Δ737 of dGB2, thus demonstrating that the intact ordered C‐terminus of dGB2 in *drosophila* GABA_B_ receptor is crucial for G proteins activation. To note, whereas the other truncated mutants of dGB2 containing an intact ordered C‐terminus (Δ734 and Δ731) show a similar effect on G protein activation as Δ737, the ability of mutant Δ728 to activate G protein is also decreased (32% reduction in *E*_max_) (Figure 5d; Figure S9e,f, Table S5, Supporting Information). Although disordered in the inactive state, the ordered C‐terminus of dGB2 is resolved up to residue 717–728 in the G_i_‐coupled activate state, which is in line with our finding that residues between 717 and 728 are crucial for *drosophila* GABA_B_ receptor activation.

We observed that the residue Q718 in the ordered C‐terminus of dGB2 forms a hydrogen bond with residue Q475 in the ICL1 of dGB2, and the residue T728 in the ordered C‐terminus of dGB2 stays close to residue R476 in the ICL1 of dGB2 (Figure 5a), which is also involved in the G protein binding pocket interacting with residue F354 in the α5 helix of G_i1_ (Figure 4f). To investigate whether these interactions were involved in G protein coupling, we replaced each of these residues with alanine to alter their side‐chain features (Q475A, Q718A, and T728A). The cell surface expression levels of all mutants co‐expressed with wild‐type dGB1 in HEK293 cells were similar to those of the wild‐type *drosophila* GABA_B_ receptor (Figure S9g, Table S5, supporting information). dGB2^Q475A^ reduced G protein activation (27% reduction in *E*_max_, *P* = 0.011), whereas dGB2^Q718A^ did not significantly reduce G protein activation (Figure 5e; Figure S9h, Table S5, Supporting Information), indicating that the hydrogen bond between the C‐terminus and ICL1 is important for G protein activation. Furthermore, dGB2^T728A^ affected G protein activation (25% reduction in *E*_max_, *P* = 0.0154) (Figure 5e; Figure S9h, Table S5, Supporting Information), demonstrating that the interaction between T728 and R476 in dGB2 is also involved in G protein coupling. Our data suggest that the ordered C‐terminus of dGB2 may interact with ICL1 to facilitate ICL1 interacting with the α5 helix of G_i1_.

To verify if the ordered C‐terminus of human GB2 is also required for G protein coupling, we generated the similar truncations of GB2 mutants after (Δ780, Δ772, and Δ765) and within (Δ760 and Δ753) the corresponding C‐terminus of human GB2 based on the sequence alignment (Figure 5b; Figure S9d, Table S5, Supporting Information). We then measured the intracellular Ca^2+^ release induced by GABA in HEK293 cells co‐transfected with GB1 mutant lacking the coiled‐coil domain (GB1^ΔCC^) and various GB2 truncated mutants. We monitored that truncated mutants of GB2 within C‐terminus (Δ760 and Δ753) largely reduce G protein activation (39% and 76% reduction in *E*_max_, 16.2‐fold and 30.8‐fold increase in EC_50_, respectively) (Figure 5f; Figure S9e,f, Table S5, Supporting Information) compared with that of Δ780 of GB2, thus demonstrating that the corresponding C‐terminus of human GB2 is also crucial for G protein activation. Interestingly, the ability of the other truncated mutants of GB2 containing an intact corresponding C‐terminus (Δ772 and Δ765) to activate G protein is also decreased (22% reduction in *E*_max_ for Δ765, 6.5‐fold and 9.4‐fold increase in EC_50_, respectively), indicating the different interaction between C‐terminus and G protein during evolution (Figure 5f; Figure S9e,f, Table S5, Supporting Information).

It should be noted that we used the constructs encoding the human heterotrimeric G protein for recombinant expression and purification of the *drosophila* GABA_B_ receptor‐G_i1_ protein complex. The α5 helix of the G_α_ protein plays a major role during the GPCR activation process.^[^ ^30^ ^]^ According to the sequence alignment and superimposed structures of G_il_ proteins in mammals (derived from the structural model of human GABA_B_ receptor) and *drosophila* (predicated by program AlphaFold2 program),^[^ ^31^ ^]^ the α5 helix is highly conserved (Figure S9i,j, Supporting Information), suggesting their physiological functions are highly similar.

## 3 Discussion

In this study, we have resolved the cryo‐EM structures of the *drosophila* GABA_B_ receptor in the CGP54626‐bound inactive state and the GABA‐bound active state in complex with the G_i1_ protein. Although *drosophila* GABA_B_ receptor also undergoes an asymmetric coupling of G_i_ protein through dGB2 as is also observed in its human homolog GB2, several major differences have been observed between *drosophila* and human GABA_B_ receptor: 1) *drosophila* GABA_B_ receptor forms a larger inactive TM3‐5/TM3‐5 interface, thus limiting its constitutive activity; 2) Four non‐conserved residues within the active 7TM interface in *drosophila* GABA_B_ receptor are the key residues responsible for PAM activity in its human homolog; 3) Whereas ICL2 is usually important in mammalian class C GPCR, ICL2 of dGB2 is less involved in G protein coupling. 4) Both the ordered C‐terminus of dGB2 and the corresponding region of its human homolog are required for G_i_ protein coupling but with some differences.

We show that the antagonist‐bound *drosophila* GABA_B_ receptor forms a larger inactive 7TM interface through TM3‐5. Previous studies have shown that the constitutive activity of the human GABA_B_ receptor is inhibited by the inter‐subunits latch located within the inactive 7TM interface.^[^ ^14^, ^26^ ^]^ In this inter‐subunit interface, although the residue in Layer I changes in dGB1‐V658^5.52^ versus GB1‐L665^5.52^, and dGB2‐V635^5.52^ versus GB2‐L669^5.52^, the residues for intensive hydrophobic contact in Layer II change in dGB1‐F659^5.53^ versus GB1‐L666^5.53^. The hydrophobic contacts in Layer I are formed through leucine and valines, and the intensive hydrophobic contacts in Layer II are formed between phenylalanine and leucine, such mutations during evolution will not alter their hydrophobic contact property. In layer III, the residues responsible for inter‐subunits latch including dGB1^H566^/dGB2^E643^ and dGB1^E666^/dGB2^H545^ are also conserved in *the drosophila* GABA_B_ receptor. These data suggest that the inactive interface formed by the close contact between TM3 and TM5 controls GABA_B_ receptor constitutive activity. Since the inactive 7TM interface is larger in the *drosophila* GABA_B_ receptor, it is obvious that such closer contact completely abolishes its constitutive activity. Our previous study demonstrated that the human GABAB receptor did not exhibit detectable constitutive activity in neurons, in contrast to what was observed in transfected cell lines.^[^ ^32^ ^]^ This might be explained by the specific tissues or cellular environment of the receptor in the brain. We presume that the reason for the absence of constitutive activity in the *drosophila* GABA_B_ receptor may be a necessity to limit the activity at the level of the receptor itself. Meanwhile, differences in the constitutive activity of GPCRs between humans and other animals may contribute to adaptive physiological responses to environmental or challenges, as we previously reported in birds.^[^ ^33^ ^]^ Given that downregulation of the *drosophila* GABA_B_ receptor in neurons has been shown to impair sleep maintenance,^[^ ^22^ ^]^ we propose that the absence of constitutive activity in the *drosophila* GABA_B_ receptor may promote increased activity and reduced sleep, thereby conferring an evolutionary advantage for survival during periods of environmental stress, such as food scarcity.

To investigate the evolutionary significance of these three layers within the TM3‐5 interface, we performed an evolutionary analysis and constructed phylogenetic trees for GABA_B_ receptors from 31 species, including *Echinodermata, Cephalochordata, Brachiopoda, Fishes, Nematomorpha, Amphibians, Reptiles*, Insects, Birds and Mammals (Figures S10,S11, Table S7, Supporting Information). We found that residues within the three layers are highly conserved across bilaterian animals, with only a few species exhibiting point mutations (Figure S12, Supporting Information). For Layer III, residues 5.60 and 3.55 are highly conserved as glutamic acid and histidine, respectively, in both the GB1 and GB2 subunits, forming a conserved salt‐bridge network that acts as the inter‐subunit latch. In contrast, residues that are involved in the interaction between position 5.52 in layer I and 5.53 in layer II of GB1 and GB2 are conserved exclusively in vertebrates but not in invertebrates. The specific residues V^5.52^ and F^5.53^ at the corresponding positions of dGB1 and dGB2 suggest their potential critical role in the constitutive activity of GABA_B_ receptors, a hypothesis that has been confirmed by our experimental results.

Previous studies have revealed that the active 7TM interface through TM6 serves as a common hallmark for the activation of class C GPCRs, including CaSR,^[^ ^28^, ^34^ ^]^ mGluRs,^[^ ^25^, ^28^, ^35^ ^]^ and the human GABA_B_ receptor.^[^ ^14^, ^36^ ^]^ Some genetic mutations in the TM6 of human GB2 lead to high constitutive activity and are associated with Rett syndrome, epileptic encephalopathy, and infantile epileptic spasms.^[^ ^5^ ^]^ Three structural studies on human GABA_B_ receptor with a PAM bound have been reported. Two of these studies examined the PAM rac‐BHFF,^[^ ^14b,c^ ^]^ while the other focused on the GS39783 (Table S8, supporting information).^[^ ^14d^ ^]^ Although the PAM binding pocket within the active 7TM interface looks similar, we demonstrate that the key residues within the pocket responsible for PAM effect are not conserved in the *drosophila* GABA_B_ receptor. Introducing these four key residues of GB1 and GB2 for PAM binding into dGB1 and dGB2 respectively restores the *drosophila* GABA_B_ receptor response to PAM, whereas replacing these four key residues with corresponding residues from the *drosophila* GABA_B_ receptor totally abolish the GABA_B_ receptor response for PAM. According to our evolutionary analysis (Figures S10,S11, Table S7, Supporting Information), the key residues in GB1 involved in PAM binding show distinct evolutionary patterns. Six of these (A^5.58^, Y^5.59^, R^6.37^, G^6.40^, M^6.41^, and Y^6.44^) are highly conserved across bilaterians, whereas I^5.55^ and K792^ICL3^ exhibit marked divergence between vertebrates and invertebrates. Specifically, I^5.55^ is substituted with L^5.55^ in invertebrates, and K792^ICL3^ is replaced by Arginine. In GB2, critical PAM‐interacting residues are largely conserved in most bilaterians except brachiopods. A functional PAM‐binding pocket formed by these key residues emerges in nematodes, but *drosophila* exhibits specific mutations at H^6.38^ (replacing Y^6.38^) and F^6.41^ (replacing M^6.41^).

Additionally, we previously developed the first NAM of the GABA_B_ receptor, CLH304a.^[^ ^8a^ ^]^ Although a prior study attempted to resolve the GABA_B_ receptor structure in complex with this NAM, the compound was not observed in the structure model.^[^ ^36^ ^]^ Both CLH304a and the PAM rac‐BHFF are derivatives of the PAM CGP7930, yet the precise binding sites of CLH304a on the GABA_B_ receptor remain unclear. Given that the rac‐BHFF binds to the TM6 interface of the GABA_B_ receptor in the active state, we propose that CLH304a also targets the TM6 interface, where it prevents the receptor from undergoing the conformational changes required for activation, thereby acting as a NAM. It will be interesting to develop the novel NAMs by targeting these four key residues of the GABA_B_ receptor to decrease the constitutive activity produced by the genetic mutations in the diseases mentioned above. Furthermore, baclofen and other synthetic agonists are less potent for insect GABA_B_ receptors such as *drosophila* and cockroaches.^[^ ^19a,b^ ^]^ Our findings may thus facilitate the development of specific PAMs for insect GABA_B_ receptors, potentially enabling their application as insecticides.

**Figure 6:**
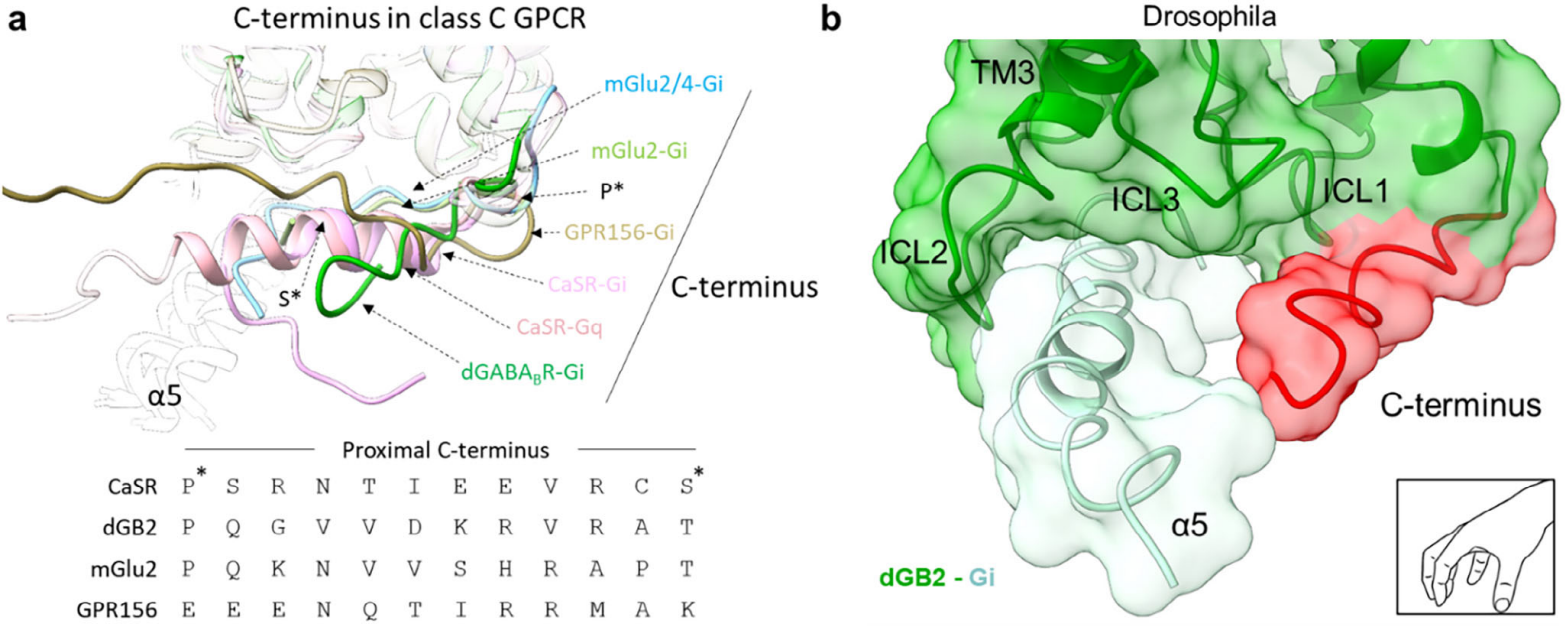
Structure basis of GABA_B_ receptor activation during evolution. a) Superimposed structures of the ordered C‐terminus in class C GPCRs, including G_i1_‐bound mGlu2 of mGlu2/4 heterodimer (PDB 8JD5, blue), G_i1_‐bound mGlu2 homodimer (PDB 7MTS, light green), G_i1_‐bound GPR156 homodimer (PDB 8YK0, brown), G_i1_‐ (PDB 8SZH, pink), and G_q_ (PDB 8SZG, orange)‐bound CaSR homodimer and *drosophila* GABA_B_ receptor heterodimer. The stars represent the positions of the indicated residues in the ordered C‐terminus of the G_q_‐bound CaSR homodimer. b) Highlight of the role of the ordered C‐terminus in the dGB2 subunit within the G_i1_ binding pocket, acting like a claw with five fingers.

Recent studies have shown that the cytoplasmic C‐terminus of GPCRs may also affect receptor function.^[^ ^28^ ^]^ Previous studies indicate that the cytoplasmic C‐terminus regions may influence the function of the GABA_B_ receptor.^[^ ^13^, ^15^ ^]^ In our study, we demonstrate that the ordered C‐terminus in dGB2 is involved in G protein coupling, whereas ICL2 is less involved. Our previous study has revealed that although the *drosophila* GABA_B_ receptor displays distinct cell surface expression patterns, the coiled‐coil domain in the C‐terminus contributes to the formation of GB1 and GB2 heterodimers at the cell surface remains conserved during evolution. Interestingly, our previous results also indicate that the ER retention signal of dGB2 is located insides the ICL2.^[^ ^24^ ^]^ The possible interaction between the coiled‐coil domain and ICL2 in dGB2 may explain why ICL2 is less involved in G protein coupling. Recent studies have revealed that the ordered C‐terminus is important for Class C GPCRs in coupling to G protein (**Figure** **6**a).^[^ ^28^, ^34^, ^35^, ^37^ ^]^ For example, the CaSR has been shown to couple to G_q_ proteins through strong interactions of the ordered C‐terminus with ICL1 and ICL3. Whereas CaSR couples to G_i_ protein mostly through interaction with all ICLs, the ordered C‐terminus is less involved.^[^ ^28b^ ^]^ We show here that the C‐terminus is also involved in human GABA_B_ receptor coupling to G_i_ protein, thus suggesting a molecular mechanism that TM3 and ICL1‐3 together with the ordered C‐terminus of GB2 form a crew with five fingers for G protein coupling (Figure 6b). Phylogenetic analysis also revealed that the G protein coupling regions of the GB2 subunit—‐including ICL1, TM3, ICL2, ICL3, and the ordered C‐terminus of the GB2 subunit—residues are highly conserved in vertebrates, but show greater diversity in invertebrates (Figures S10,S11, Table S7, Supporting Information). This suggests that invertebrate GABA_B_ receptors may adopt distinct G protein coupling mechanisms compared to vertebrate receptors. In all, our study not only provides a novel but conserved molecular basis for functions of GABA_B_ receptors during evolution but also novel insight for future GABA_B_ receptors medications and insecticides development.

## 4 Experimental Section

### Materials

GABA (Cat. HY‐N0067) was purchased from MedChemExpress (Monmouth Junction, NJ, USA). SKF97541 (Cat. 0379) and rac‐ BHFF (Cat. 3313) were purchased from Tocris Bioscience (Ellisville, MO, USA). CGP54626 (Cat. 20 121) was purchased from Cayman Chemical (Ann Arbor, MI, USA). Lipofectamine 2000 (Cat. 11 668 019) and Fluo4‐AM (Cat. M14206) were obtained from Thermo Fisher Scientific (Waltham, MA, USA). Furimazine (Cat. N1120) was from Promega (Madison, WI, USA).

### Constructs

In sample preparation for Cryo‐EM, *drosophila* GABA_B1_ (UniProt code: Q9BML7) and GABA_B2_ (UniProt code: Q9BML6) subunits were cloned into a modified pEG‐BacMam vector for co‐expression in baculovirus‐infected mammalian cells, respectively.^[^ ^38^ ^]^ Both full‐length (WT) and C‐terminal truncated (EM) forms were tested for heterodimeric assembly. The dGB1 EM construct spanned residues 29–800, and the dGB2 EM from residues 24–790. Influenza hemagglutinin signal sequences replaced the native signals,^[^ ^39^ ^]^ removing 28 residues from dGB1 and 23 from dGB2. A 3C protease cleavage site and 8× histidine tag were added to dGB1, and a FLAG‐tag and 2× GSG linker to dGB2 for purification. All the constructs were confirmed by sequencing.

For G‐protein signalling assays, the pRK5 plasmids encoding HA‐tagged wild‐type human GB1a, dGB1, and FLAG‐tagged wild‐type human GB2 were used for signaling assays, as previously described.^[^ ^14^, ^24^ ^]^ Each construct included the plasma membrane signal peptide of metabotropic glutamate receptor 5 at the N‐terminus. For the C‐terminal truncated constructs, residue 748 of dGB1 and residue 881 of GB1 were substituted by a stop codon in the PRK5 vector and were denoted as dGB1^ΔCC^ and GB1^ΔCC^, respectively. Similarly, residue 738, 735, 732, 729, 727, 724, 721, and 718 of dGB2 were substituted by a stop codon in PEG‐BacMam vector and were denoted as Δ737, Δ734, Δ731, Δ728, Δ726, Δ723, Δ720, and Δ717, respectively. Notably, these dGB2 C‐terminal truncations use the influenza hemagglutinin signal peptide to facilitate its cell surface expression, even in the absence of the coiled‐coil domain. Residues 781, 773, 766, 761, and 754 of human GB2 were replaced by a stop codon in the PRK vector, generating the Δ780, Δ772, Δ765, Δ760, and Δ753, respectively. These mutants were creating using site‐directed mutagenesis with the Quick‐change mutagenesis protocol (Agilent Technologies).

HEK293 cells (ATCC, CRL‐1573) were cultured in DMEM with 10% fetal bovine serum at 37 °C and 5% CO_2_, and tested negative for mycoplasma contamination. Cells were plated at 0.5 million cells mL^−1^ in 6‐well plates and co‐transfected with WT (HA–dGB1 and FLAG–dGB2) or mutant constructs using Lipofectamine 2000 in 200 µL Opti‐MEM (Thermo Fisher Scientific). For intracellular calcium measurements, cells were also transfected with the chimeric G‐protein Gqi9 to couple the recombinant GABAB receptor to phospholipase C.^[^ ^11c^ ^]^ Functional assays and cell‐surface expression were performed after 24 h of incubation (14 h at 37 °C, 5% CO_2_ followed by 10 h at 30 °C, 5% CO_2_).

### Expression and Purification of Inactive Drosophila GABA_B_ Receptor

HEK293F cells were cultured in suspension at 37 °C in 5% CO_2_ using SMM 293‐TI medium (Sino Biological, Beijing, CN). The cells were co‐infected with recombinant baculoviruses carrying the dGB1 and dGB2 genes at a 1:1 ratio. To enhance expression levels, 15 mm sodium butyrate was added 20 h post‐infection, and the cells were incubated for an additional 72 h at 30 °C before harvesting.

For the purification of inactive *drosophila* GABA_B_ receptor, HEK293F cell pellets were lysed using a KIMBLE Dounce homogenizer in buffer containing 50 mm HEPES (pH 7.5), 150 mm NaCl, 2 mm MgCl2, 10% glycerol, 40 µm CGP54626, and a protease inhibitor cocktail. The membrane complex was extracted by adding 0.5% lauryl maltose neopentyl glycol and 0.1% cholesteryl hemisuccinate, followed by 3 h of incubation at 4 °C. After centrifugation (36 000 × g, 50 min), the complex was purified using a Ni‐NTA column and M1 anti‐FLAG affinity resin. The sample was further purified by size exclusion chromatography on a Superose 6 Increase column (GE Healthcare) to obtain a homogeneous CGP54626‐bound *drosophila* GABA_B_ receptor complex. The final complex was concentrated to ≈2 mg mL^−1^ for single‐particle cryo‐EM sample preparation.

### Preparation of Drosophila GABA_B_‐G_i1_Gβ_1_Gγ_2_‐scFv16 Complex

ScFv16 and Heterotrimeric G_i1_ were expressed and purified as previously described.^[^ ^40^ ^]^ A carboxy‐terminal 8× His‐tagged scFv16 was cloned into the pFastBac1 vector with a GP67 secretion signal. This construct was expressed in *Trichoplusia ni* Hi5 insect cells for 48–60 h using the Bac‐to‐Bac system, followed by Ni‐NTA chromatography and gel filtration chromatography using a Superdex 200 column for purification. The purified scFv16 was concentrated and stored at −80 °C for further use.

For G_i1_, the dominant‐negative Gα_i1_ mutants (S47N, G203A, E245A and A326S) along with human β_1_γ_2_ subunits (β1–6× His tag) were co‐expressed in Hi5 insect cells using the Bac‐to‐Bac system. Cells were harvested after 60 h of expression, flash‐frozen in liquid nitrogen, and stored at −80 °C for further use.

For the purification of *drosophila* GABA_B_‐Gα_i1_ complex, Hi5 cells expressing heterotrimeric Gi1 were lysed in a buffer containing 10 mm HEPES (pH 7.5), 0.1 mm MgCl2, 0.1 mm TCEP, 0.01 mm GDP, and protease inhibitors. The membranes were collected by centrifugation. Both Hi5 cell membranes and HEK293F cell pellets were then lysed in a buffer with 50 mm HEPES, 150 mm NaCl, 2 mm MgCl2, 10% glycerol, and protease inhibitors. The sample was incubated at room temperature for 2 h with 300 µm GABA, 25 mU mL^−1^ apyrase, and 10 µg mL^−1^ scFv16. The complex was extracted using 0.5% lauryl maltose neopentyl glycol and 0.1% cholesteryl hemisuccinate, incubated for 3 h at 4 °C, and then centrifuged at 36 000 × g for 50 min. The complex was purified via Ni‐NTA chromatography, followed by M1 anti‐FLAG resin, and further purified using a Superose 6 Increase column (GE Healthcare) by size exclusion chromatography. The final complex was concentrated to ≈2 mg mL^−1^ for cryo‐EM analysis.

### Single‐Particle Cryo‐EM Sample Preparation and Data Collection

For cryo‐EM grid preparation, 3 µL of purified antagonist bound *drosophila* GABA_B_ heterodimer or GABA‐bound *drosophila* GABA_B_–G_i1_ complex (1.8 mg mL^−1^) was applied to glow‐discharged holey carbon grids (Quantifoil, R1.2/1.3, 300 mesh). The grids were blotted for 3 s with a blot force of 3 at 4 °C and 100% humidity, followed by plunge‐frozen in liquid ethane using a Vitrobot Mark IV (Thermo Fischer Scientific). Cryo‐EM data collection was collected on a Titan Krios operating at 300 kV in the center of cyro‐EM. Micrographs were recorded using a Gatan K3 Summit Detector in counting mode with a pixel size of 0.93 Å, controlled by SerialEM software.^[^ ^41^ ^]^ Image stacks were acquired at a dose rate of ≈7 electrons per Å^2^ per second with a defocus ranging from −1 to −2.5 µm. Each exposure lasted 6 s, acquiring 40 frames per micrograph. A total of 7541 and 15 775 movies were collected for the inactive state *drosophila* GABA_B_ and GABA_B_–G_i1_ complex, respectively.

### Cryo‐EM Image Processing

Image stacks for the antagonist‐bound *drosophila* GABA_B_ heterodimer and GABA‐bound *drosophila* GABA_B_–G_i1_ complex were processed by CryoSPARC software (v 4.4.0).^[^ ^42^ ^]^ After patch motion correction and CTF estimation, particles were picked via Blob picker and refined through 2D classification. Initial models were generated using a stochastic gradient descent algorithm, followed by heterogeneous refinement to isolate distinct conformational states. Final refinement of the heterodimer maps was performed with CryoSPARC's non‐uniform and local refinement (masked to include only VFTs, 7TMs, or G‐protein). Composite maps were generated in Chimera (v1.15),^[^ ^43^ ^]^ sharpened in CryoSPARC, and validated with Relion.^[^ ^44^ ^]^ Model validation was performed using Phenix (v.1.20.1‐4487). with visualizations and figure preparation in UCSF Chimera and Chimera X (v1.5, v1.6.1).

### Model Building and Refinement

The initial model of the *drosophila* GABA_B_ heterodimer was based on the AlphaFold‐predicted structure, while the Gi1 and scFv16 coordinates were derived from the human cannabinoid receptor 2–Gi1 complex (PDB 6PT0).^[^ ^45^ ^]^ Models of GABA_B_ and GABA_B_–Gi1–scFv16 were docked into the cryo‐EM map using UCSF Chimera. Agonist and antagonist coordinates were generated with phenix.elbow. The docked model underwent flexible fitting with Rosetta, followed by rebuilding in Coot and real‐space refinement in Rosetta and Phenix. Final refinement was validated with the “comprehensive validation (cryo‐EM)” module in Phenix, and model‐map fit was assessed using Fourier shell correlation. Refinement statistics are presented in Table 1. Structural figures were prepared using UCSF Chimera and Chimera X.

### Cell Surface Quantification by ELISA

Cell surface expression of WT or mutant constructs was assessed by ELISA as previously described.^[^ ^24^ ^]^ HEK293 cells were transfected and seeded into 96‐well plates, then incubated for 24 h. After fixation with 4% paraformaldehyde and blocking with 1% FBS, HA‐tagged constructs were detected using a rat anti‐HA antibody (3F10, Roche) conjugated to horseradish peroxidase (HRP). FLAG‐tagged constructs were detected with a mouse anti‐FLAG antibody (A8592, Sigma‐Aldrich) also conjugated to HRP. Chemiluminescence was measured using Super Signal ELISA Femto Maximum Sensitivity substrate (Thermo Fisher Scientific) on a 2103 EnVisionTM Multilabel Plate Reader (Perkin Elmer, Waltham, MA, USA).

### Intracellular Calcium Release Measurements

HEK293 cells were transfected and seeded into 96‐well plates 24 h prior. After washing with HBSS buffer (20 mm HEPES pH 7.4, 1 mm MgSO_4_, 3.3 mm Na_2_CO_3_, 1.3 mm CaCl_2_, 0.1% BSA, and 2.5 mm probenecid), cells were loaded with 1 µm Fluo‐4 AM (Thermo Fisher Scientific) for 1 h at 37 °C. Following one wash with HBSS buffer, 50 µL of buffer was added to each well, followed by 50 µL of 2× GABA solution at various concentrations after 40 s of recording. Fluorescence (excitation: 485 nm, emission: 525 nm) was measured for 120 s using a FLIPR Tetra fluorescence microplate reader (Molecular Devices, Sunnyvale, CA, USA). Dose‐response curves were generated and fitted using GraphPad Prism software.

### Inositol Phosphate (IP_1_) Measurements

IP_1_ accumulation was measured using the IP‐One HTRF kit (62IPAPEJ, Revvity) in 96‐well plates. Twenty‐four hours after transfection, HEK293 cells were washed, treated with stimulation buffer for 45 min at 37 °C, and then incubated with d2‐labeled IP_1_ (IP_1_‐d2) and terbium cryptate‐labeled anti‐IP_1_ antibody (Anti‐IP_1_‐K) in lysis buffer. After 1 h in the dark at room temperature, fluorescence was measured using a PHERAstar FSX plate reader (BMG LABTECH) with excitation at 337 nm and emissions at 620 and 665 nm. Results were calculated based on the fluorescence ratio of IP_1_‐d2 emission at 665 nm to Anti‐IP_1_‐K emission at 620 nm, using the standard curve.

### BRET Measurement

HEK293 cells were transfected with the GABA_B_ receptor, the Gα_i1_‐case plasmid (BRET sensor),^[^ ^29^ ^]^ then split into 96‐well microplates. After 24 h, cells were washed and starved in PBS at 37 °C for 30 min. BRET measurements were performed using the multi‐mode plate reader after adding GABA at varying concentrations. The BRET signal was calculated as the ratio of emissions at 535 and 475 nm (integration time 0.1 s). The ΔBRET ratio was determined as the difference between the experimental BRET signal and the basal BRET ratio, recorded before GABA stimulation.

### Statistical Analysis

The raw data of the intracellular calcium release measurements, BRET, and ELISA assay were standardized by the corresponding WT group, while the raw data of the IP_1_ accumulation assay were standardized by the corresponding Mock group. For dose–response experiments, data were normalized and analyzed using nonlinear curve fitting for the log (agonist) versus response (four parameters) curves by GraphPad Prism (GraphPad Software, San Diego, CA, USA). Data were shown as mean ± SEM from at least three independent experiments, performed in triplicates. Bars represent differences in the calculated agonist potency (pEC_50_), and maximum agonist response (*E*_max_) for each mutant relative to the wild‐type group. Statistical analyses were conducted by GraphPad Prism using a one‐way analysis of variance (ANOVA) test followed by a Dunnett's multiple comparison test. For the comparison only containing two members, the unpaired Student's *t*‐test (two‐tailed) was performed. Nd, not determined. Ns, not significant. Statistical significance was set at p < 0.05, with details provided in the figure legends. *P < 0.05, **P < 0.01, ***P < 0.001, ****P < 0.0001.

## Conflict of Interest

The authors declare no conflict of interest.

## Author Contributions

G.H., S.Z., C.S., S.J., and B.Z. contributed equally to this work. J.H., Y.Z., and J.L. conceived and supervised the whole project. G.H. and S.Z. designed the constructs and expressed and purified the recombinant proteins. C.S., S.J., and B.Z. prepared the cryo‐EM grids, collected the cryo‐EM data, and performed Cryo‐map calculation and model building. G.H. and L.L. performed functional assays. D.S. and J.L. evaluated samples by negative‐stain electron microscopy. G.H., S.Z., and C.S. prepared the figures. G.H., S.Z., and C.S. participated in manuscript writing. C.X. and P.R. participated in the interpretation of the data and the preparation of the manuscript. S.Z. and J.L. wrote the manuscript with input from all the authors.

## Supporting information



Supporting Information

## Data Availability

All data generated in this study are included in the main text or the Supplementary Information. Cryo‐EM maps have been deposited in the Protein Data Bank and Electron Microscopy Data Bank under accession codes: GABA‐DGABA_B_R‐Gi‐scfv16: PDB 9JQX, EMDB 61742; CGP54626‐DGABA_B_R: PDB 9JR0, EMDB 61745.

## References

[1] a) M. Terunuma , Proc. Jpn. Acad., Ser. B 2018, 94, 390; 30541966 10.2183/pjab.94.026 PMC6374141

[2] G. Chen , A. N. van den Pol , J. Neurosci. 1998, 18, 1913. 9465016 10.1523/JNEUROSCI.18-05-01913.1998 PMC6792632

[3] D. L. Sodickson , B. P. Bean , J. Neurosci. 1996, 16, 6374. 8815916 10.1523/JNEUROSCI.16-20-06374.1996 PMC6578909

[4] a) M. Gassmann , B. Bettler , Nat. Rev. Neurosci. 2012, 13, 380; 22595784 10.1038/nrn3249

[5] a) Y. Yoo , J. Jung , Y. N. Lee , Y. Lee , H. Cho , E. Na , J. Hong , E. Kim , J. S. Lee , J. S. Lee , C. Hong , S. Y. Park , J. Wie , K. Miller , N. Shur , C. Clow , R. S. Ebel , S. D. DeBrosse , L. B. Henderson , R. Willaert , C. Castaldi , I. Tikhonova , K. Bilguvar , S. Mane , K. J. Kim , Y. S. Hwang , S. G. Lee , I. So , B. C. Lim , H. J. Choi , et al., Ann. Neurol. 2017, 82, 466; 28856709 10.1002/ana.25032

[6] a) Y. Sammaraiee , M. Yardley , L. Keenan , K. Buchanan , V. Stevenson , R. Farrell , Mult. Scler. Relat. Disord. 2019, 27, 95; 30366276 10.1016/j.msard.2018.10.009

[7] a) K. Zemoura , W. T. Ralvenius , P. Malherbe , D. Benke , Neuropharmacology 2016, 108, 172; 27108932 10.1016/j.neuropharm.2016.04.028

[8] a) B. Sun , L. Chen , L. Liu , Z. Xia , J. P. Pin , F. Nan , J. Liu , Biochem. J. 2016, 473, 779; 26772870 10.1042/BJ20150979

[9] C. M. Niswender , P. J. Conn , Annu. Rev. Pharmacol. Toxicol. 2010, 50, 295. 20055706 10.1146/annurev.pharmtox.011008.145533 PMC2904507

[10] J. Kniazeff , L. Prezeau , P. Rondard , J. P. Pin , C. Goudet , Pharmacol. Ther. 2011, 130, 9. 21256155 10.1016/j.pharmthera.2011.01.006

[11] a) K. Kaupmann , B. Malitschek , V. Schuler , J. Heid , W. Froestl , P. Beck , J. Mosbacher , S. Bischoff , A. Kulik , R. Shigemoto , A. Karschin , B. Bettler , Nature 1998, 396, 683; 9872317 10.1038/25360

[12] a) B. Malitschek , C. Schweizer , M. Keir , J. Heid , W. Froestl , J. Mosbacher , R. Kuhn , J. Henley , C. Joly , J. P. Pin , K. Kaupmann , B. Bettler , Mol. Pharmacol. 1999, 56, 448; 10419566 10.1124/mol.56.2.448

[13] a) M. Margeta‐Mitrovic , Y. N. Jan , L. Y. Jan , Proc. Natl. Acad. Sci. USA 2001, 98, 14649; 11724956 10.1073/pnas.251554498 PMC64736

[14] a) L. Xue , Q. Sun , H. Zhao , X. Rovira , S. Gai , Q. He , J. P. Pin , J. Liu , P. Rondard , Nat. Commun. 2019, 10, 2765; 31235691 10.1038/s41467-019-10834-5 PMC6591306

[15] A. M. Pooler , A. G. Gray , R. A. McIlhinney , Eur. J. Neurosci. 2009, 29, 869. 19291218 10.1111/j.1460-9568.2009.06636.x

[16] a) A. Couve , A. K. Filippov , C. N. Connolly , B. Bettler , D. A. Brown , S. J. Moss , J. Biol. Chem. 1998, 273, 26361; 9756866 10.1074/jbc.273.41.26361

[17] S. Burmakina , Y. Geng , Y. Chen , Q. R. Fan , Proc. Natl. Acad. Sci. USA 2014, 111, 6958. 24778228 10.1073/pnas.1400081111 PMC4024898

[18] a) R. A. Kammerer , S. Frank , T. Schulthess , R. Landwehr , A. Lustig , J. Engel , Biochemistry 1999, 38, 13263; 10529199 10.1021/bi991018t

[19] a) M. Mezler , T. Muller , K. Raming , Eur. J. Neurosci. 2001, 13, 477; 11168554 10.1046/j.1460-9568.2001.01410.x

[20] a) L. Chun , J. Gong , F. Yuan , B. Zhang , H. Liu , T. Zheng , T. Yu , X. Z. Xu , J. Liu , Nat. Commun. 2015, 6, 8828; 26537867 10.1038/ncomms9828 PMC4667614

[21] Y. Hamasaka , C. Wegener , D. R. Nassel , J. Neurobiol. 2005, 65, 225. 16118795 10.1002/neu.20184

[22] F. Gmeiner , A. Kolodziejczyk , T. Yoshii , D. Rieger , D. R. Nassel , C. Helfrich‐Forster , J. Exp. Biol. 2013, 216, 3837. 24068350 10.1242/jeb.085563

[23] a) S. Dzitoyeva , A. Gutnov , M. Imbesi , N. Dimitrijevic , H. Manev , Brain Res. Dev. Brain Res. 2005, 158, 111; 16054235 10.1016/j.devbrainres.2005.06.005

[24] S. Zhang , L. Xue , X. Liu , X. C. Zhang , R. Zhou , H. Zhao , C. Shen , J. P. Pin , P. Rondard , J. Liu , FASEB J. 2020, 34, 16348. 33058267 10.1096/fj.202001355RR

[25] a) E. Jeong , Y. Kim , J. Jeong , Y. Cho , Nat. Commun. 2021, 12, 6805; 34815401 10.1038/s41467-021-27147-1 PMC8611064

[26] J. Park , Z. Fu , A. Frangaj , J. Liu , L. Mosyak , T. Shen , V. N. Slavkovich , K. M. Ray , J. Taura , B. Cao , Y. Geng , H. Zuo , Y. Kou , R. Grassucci , S. Chen , Z. Liu , X. Lin , J. P. Williams , W. J. Rice , E. T. Eng , R. K. Huang , R. K. Soni , B. Kloss , Z. Yu , J. A. Javitch , W. A. Hendrickson , P. A. Slesinger , M. Quick , J. Graziano , H. Yu , et al., Nature 2020, 584, 304. 32581365 10.1038/s41586-020-2452-0 PMC7725281

[27] J. Gomeza , C. Joly , R. Kuhn , T. Knopfel , J. Bockaert , J. P. Pin , J. Biol. Chem. 1996, 271, 2199. 8567679 10.1074/jbc.271.4.2199

[28] a) J. Perroy , G. J. Gutierrez , V. Coulon , J. Bockaert , J. P. Pin , L. Fagni , J. Biol. Chem. 2001, 276, 45800; 11584003 10.1074/jbc.M106876200

[29] H. Schihada , R. Shekhani , G. Schulte , Sci. Signaling 2021, 14, abf1653. 10.1126/scisignal.abf1653 34516756

[30] a) E. P. Marin , A. G. Krishna , T. P. Sakmar , J. Biol. Chem. 2001, 276, 27400; 11356823 10.1074/jbc.C100198200

[31] J. Jumper , R. Evans , A. Pritzel , T. Green , M. Figurnov , O. Ronneberger , K. Tunyasuvunakool , R. Bates , A. Zidek , A. Potapenko , A. Bridgland , C. Meyer , S. A. A. Kohl , A. J. Ballard , A. Cowie , B. Romera‐Paredes , S. Nikolov , R. Jain , J. Adler , T. Back , S. Petersen , D. Reiman , E. Clancy , M. Zielinski , M. Steinegger , M. Pacholska , T. Berghammer , S. Bodenstein , D. Silver , O. Vinyals , et al., Nature 2021, 596, 583. 34265844 10.1038/s41586-021-03819-2 PMC8371605

[32] C. Xu , Y. Zhou , Y. Liu , L. Lin , P. Liu , X. Wang , Z. Xu , J. P. Pin , P. Rondard , J. Liu , Nat. Commun. 2024, 15, 1990. 38443355 10.1038/s41467-024-46177-z PMC10914727

[33] C. Zhang , X. Xiang , J. Liu , Y. Huang , J. Xue , Q. Sun , S. Leng , S. Liu , X. He , P. Hu , X. Zhan , Q. Qiu , S. Yang , J. Brosius , C. Deng , Nature 2025, 641, 1287. 40031956 10.1038/s41586-025-08811-8 PMC12119371

[34] a) Y. Gao , M. J. Robertson , S. N. Rahman , A. B. Seven , C. Zhang , J. G. Meyerowitz , O. Panova , F. M. Hannan , R. V. Thakker , H. Brauner‐Osborne , J. M. Mathiesen , G. Skiniotis , Nature 2021, 595, 455; 34194040 10.1038/s41586-021-03691-0 PMC8826748

[35] a) A. Koehl , H. Hu , D. Feng , B. Sun , Y. Zhang , M. J. Robertson , M. Chu , T. S. Kobilka , T. Laeremans , J. Steyaert , J. Tarrasch , S. Dutta , R. Fonseca , W. I. Weis , J. M. Mathiesen , G. Skiniotis , B. K. Kobilka , Nature 2019, 566, 79; 30675062 10.1038/s41586-019-0881-4 PMC6709600

[36] Y. Kim , E. Jeong , J. H. Jeong , Y. Kim , Y. Cho , J. Mol. Biol. 2020, 432, 5966. 33058878 10.1016/j.jmb.2020.09.023

[37] X. Ma , L. N. Chen , M. Liao , L. Zhang , K. Xi , J. Guo , C. Shen , D. D. Shen , P. Cai , Q. Shen , J. Qi , H. Zhang , S. K. Zang , Y. J. Dong , L. Miao , J. Qin , S. Y. Ji , Y. Li , J. Liu , C. Mao , Y. Zhang , R. Chai , Nat. Commun. 2024, 15, 10601. 39638804 10.1038/s41467-024-54681-5 PMC11621567

[38] A. Goehring , C. H. Lee , K. H. Wang , J. C. Michel , D. P. Claxton , I. Baconguis , T. Althoff , S. Fischer , K. C. Garcia , E. Gouaux , Nat. Protoc. 2014, 9, 2574. 25299155 10.1038/nprot.2014.173 PMC4291175

[39] X. M. Guan , T. S. Kobilka , B. K. Kobilka , J. Biol. Chem. 1992, 267, 21995. 1331042

[40] A. Koehl , H. Hu , S. Maeda , Y. Zhang , Q. Qu , J. M. Paggi , N. R. Latorraca , D. Hilger , R. Dawson , H. Matile , G. F. X. Schertler , S. Granier , W. I. Weis , R. O. Dror , A. Manglik , G. Skiniotis , B. K. Kobilka , Nature 2018, 558, 547. 29899455 10.1038/s41586-018-0219-7 PMC6317904

[41] D. N. Mastronarde , J. Struct. Biol. 2005, 152, 36. 16182563 10.1016/j.jsb.2005.07.007

[42] A. Punjani , J. L. Rubinstein , D. J. Fleet , M. A. Brubaker , Nat. Methods 2017, 14, 290. 28165473 10.1038/nmeth.4169

[43] A. Punjani , D. J. Fleet , J. Struct. Biol. 2021, 213, 107702. 33582281 10.1016/j.jsb.2021.107702

[44] S. H. Scheres , Methods Enzymol. 2016, 579, 125. 27572726 10.1016/bs.mie.2016.04.012

[45] C. Xing , Y. Zhuang , T. H. Xu , Z. Feng , X. E. Zhou , M. Chen , L. Wang , X. Meng , Y. Xue , J. Wang , H. Liu , T. F. McGuire , G. Zhao , K. Melcher , C. Zhang , H. E. Xu , X. Q. Xie , Cell 2020, 180, 645. 32004460 10.1016/j.cell.2020.01.007 PMC8247115

