# Supplementary material for: Structural Basis of GABAB Receptor Activation during Evolution
Source: Adv Sci (Weinh). 2025 Jul 12;12(37):e09440. doi: 10.1002/advs.202509440 (PMC12499500; doi:10.1002/advs.202509440)
Supplement: Supplementary file 1 — Supporting Information [file ADVS-12-e09440-s001.docx]

Supporting Information

Structural basis of GABA_B_ receptor activation during evolution

Guofei Hou, Shenglan Zhang, Cangsong Shen, Suyu Ji, Binqian Zou, Chanjuan Xu, Liang Li, Dandan Shen, Jiayin Liang, Haidi Chen, Philippe Rondard, Cheng Deng, Jun He, Yan Zhang, and Jianfeng Liu


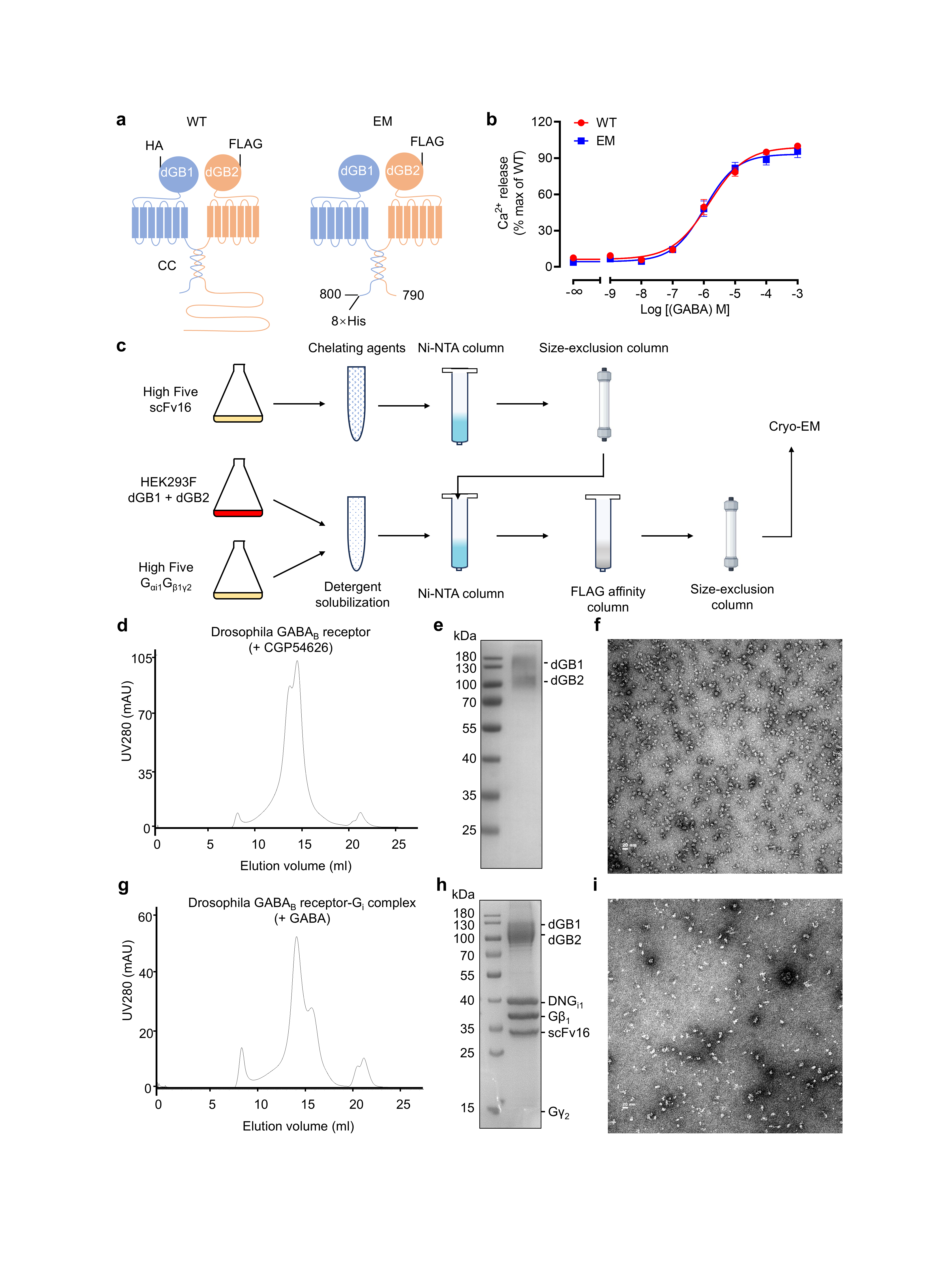


**Figure S1. Expression and purification of *drosophila* GABA_B_ receptor heterodimer and *drosophila* GABA_B_ receptor-G_i1_ complex.**

**(a)** Schematic diagram of constructs used in this study. Wild-type (WT): full-length constructs used for functional experiments. EM: Truncated constructs for Cryo-EM samples preparation. CC: coiled-coil domain. 8×His: consecutive 8 histidine. **(b)** Intracellular calcium release induced by GABA in HEK293 cells overexpressing the indicated constructs. Data are normalized by the wild-type response and shown as means ± SEM of at least three biologically independent experiments, performed in technical duplicate. **(c)** The purification scheme for *drosophila* GABA_B_ receptor heterodimer. DGB1 and dGB2 subunits were co-expressed in HEK293F cells. Heterotrimeric G_i1_Gβ_1_γ_2_ and scFv16 were expressed in Hi5 cells, respectively. **(d–f)** Size-exclusion chromatography profile *(d)*, SDS-PAGE *(e)* and the negative-staining electron microscopy analysis *(f)* of the purified antagonist CGP54626-bound *drosophila* GABA_B_ receptor. **(g-i)** Size-exclusion chromatography profile *(g)*, SDS-PAGE *(h)* and the negative-staining electron microscopy analysis *(i)* of the purified agonist GABA-bound *drosophila* GABA_B_ receptor in complex with heterotrimeric G proteins.


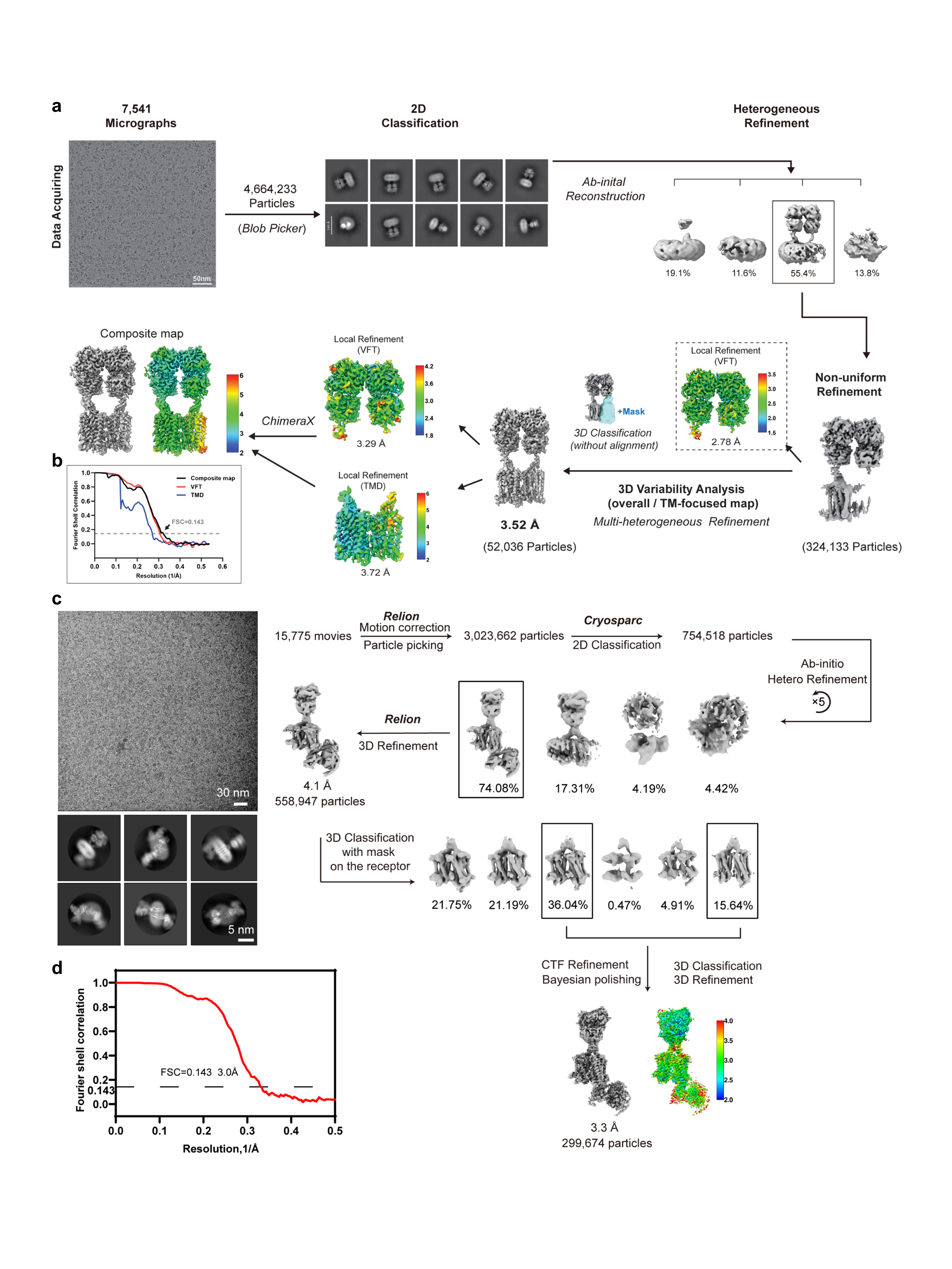


**Figure S2. Cryo-EM images and single particle reconstruction of the EM map of d*rosophila* GABA_B_R in active and inactive state.**

**(a)** Representative cryo-EM micrograph and two-dimensional class averages of the overall refined *drosophila* GABA_B_ receptor in CGP54626-bound inactive state, and flow chart of cryo-EM data processing. Details are described in method section. **(b)** Gold-standard Fourier shell correlation curves of overall refined composite receptor and the locally refined VFT and TMD. **(c)** Cryo-EM micrograph and representative two-dimensional class averages of the *drosophila* GABA_B_ receptor in GABA–bound active state complexed with G proteins in LMNG detergent micelles. Flow chart of cryo-EM data processing. Details are described in Method section. **(d)** Gold-standard Fourier shell correlation curves of overall refined *drosophila* GABA_B_-G_i1_ complex.


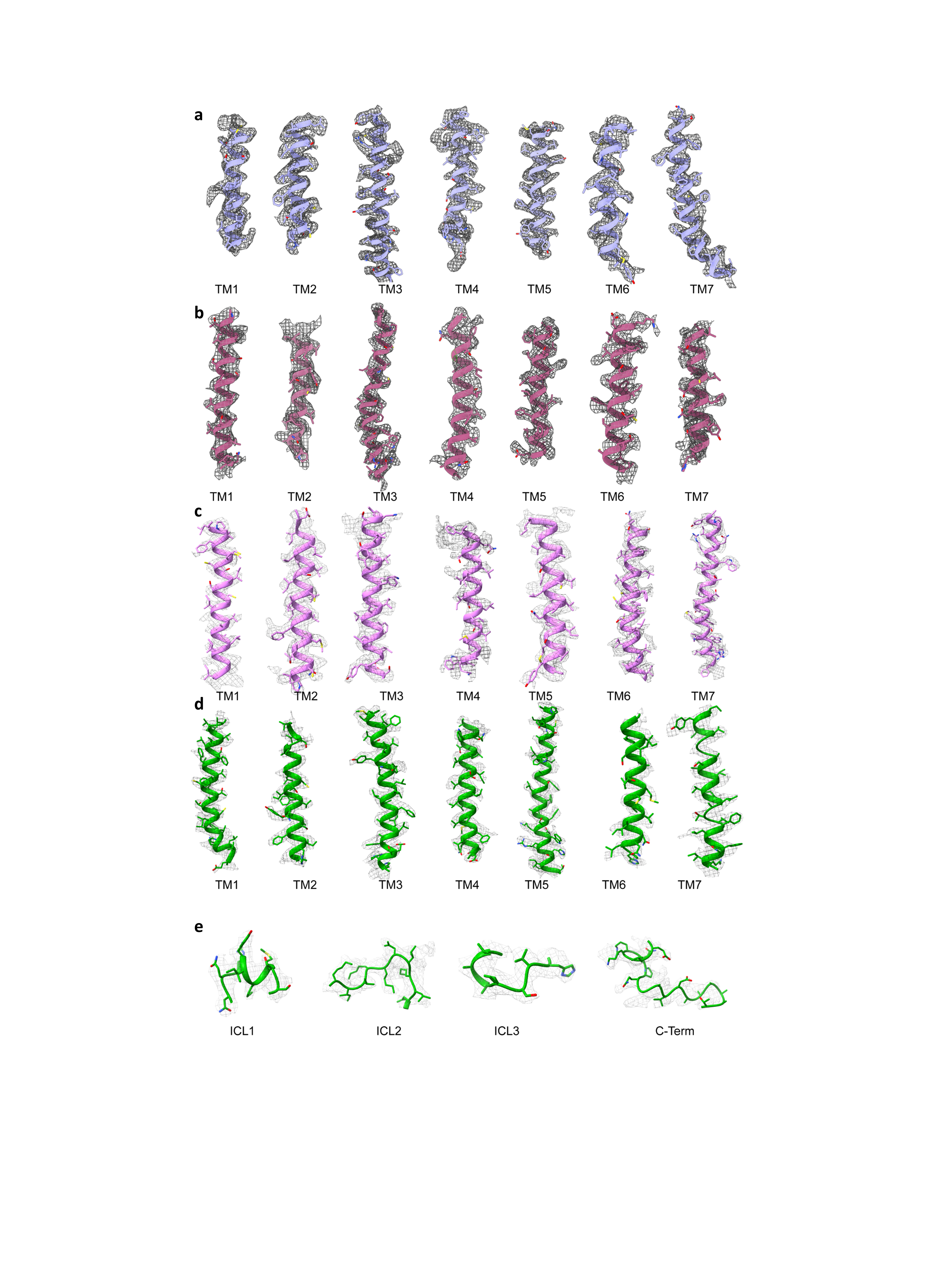


**Figure S3. Cryo-EM density analysis of *drosophila* GABA_B_ receptors.**

**(a–e)** Depicts the representative resolution map of TM1-TM7 within the inactive dGB1 *(a)*, inactive dGB2 *(b)*, active dGB1 *(c)* and active dGB2 *(d)*, respectively, and the ordered C-terminus and ICL1-3 within the active dGB2 *(e)*.


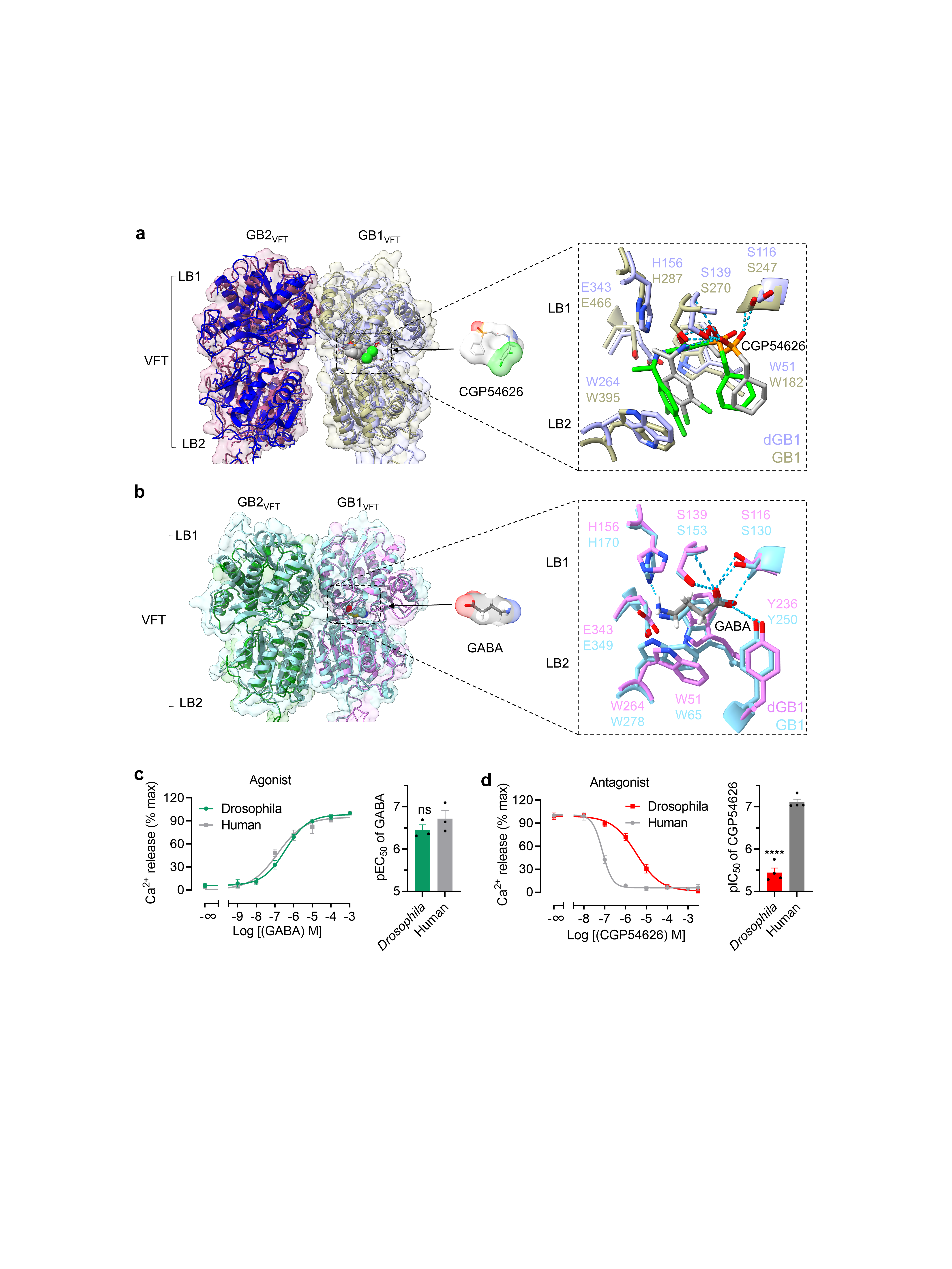


**Figure S4. The ligand-binding pockets of *drosophila* GABA_B_ receptor**

**(a–b)** Structural comparisons of extracellular VFT domains of *drosophila* and human GABA_B_ receptors in agonist CGP65626-bound form *(a)* and in agonist GABA-bound form *(b)*. The binding pockets of CGP54626 and GABA are enlarged on the right. **(c–d)** Intracellular calcium release induced by agonist GABA *(c)*, or antagonist CGP54626 in the presence of EC_80_ GABA *(d)* in HEK293 cells transfected with GABA_B_ receptors as indicated. Values are mean ± SEM. from three independent experiments, performed in technical triplicate and analyzed using the unpaired t-test (two-tailed). Data are normalized by the max response.


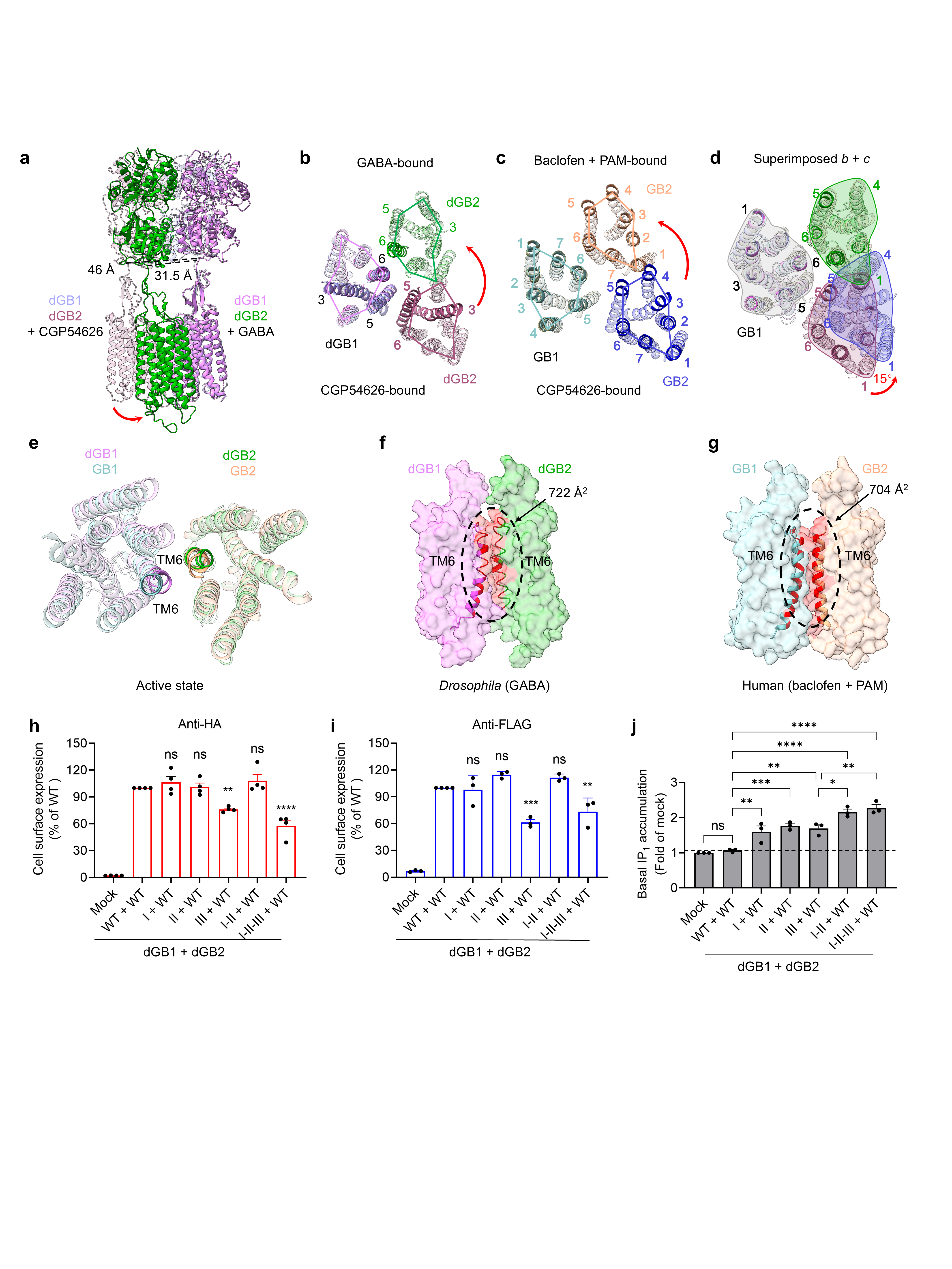


**Figure S5. Structural comparison of transmembrane regions of *drosophila* and human GABA_B_ receptors.**

**(a-b)** Side review *(a)* and top views *(b)* of the superimposed structures of *drosophila* GABA_B_ receptor in CGP54626-bound inactive state and GABA-bound active state, aligned by dGB1 subunit. The distances between the C-termini of dGB1 (residue D453) and dGB2 subunits (residue D432) in inactive and active states indicated by the Cα of the proximal residue are measured and indicated. **(c)** Top views of the superimposed structures of the human GABA_B_ receptor in inactive state and activate, aligned by GB1 subunit. **(d)** Top views of the superimposed structures of the indicated *drosophila* and human GABA_B_ receptors in different states, aligned by GB1 subunit. **(e)** Overlay of the structures of *drosophila* and human GABA_B_ receptors 7TM domains in active state. **(f–g)** Surface representations of 7TM domains of *drosophila* GABA_B_ receptor in active state *(f)*, human GABA_B_ receptor in agonist baclofen and PAM-bound active state *(g)*, the interaction areas between 7TMs were highlighted in red. PAM: rac-BHFF. NAM: CLH304a. **(h-i)** ELISA measurement of the amount of indicated HA-tagged dGB1 *(h)* and FLAG-tagged dGB2 *(i).* HEK293 cells were transfected with the indicated constructs. WT: wild-type dGB1 or dGB2. I: dGB1 mutant V658L. II: dGB1 mutant F659L. III: dGB1 mutant E666A and H566A. I-II: dGB1 mutant V658L and F659L. I-II-III: dGB1 mutant V658L, F659L, E666A and H566A. Data are normalized by wild-type response. **(j)** Basal IP_1_ accumulation for human and *drosophila* GABA_B_ receptors. HEK293 cells were transfected with the indicated constructs. Values in *(h-j)* are mean ± SEM from at least three independent experiments, performed in technical duplicate and analyzed using one-way analysis of variance with Dunnett’s multiple comparison test to determine significance (compared with wild-type or dGB1^III^). ns: not significant. Data in *(h-i)* are normalized by the wild-type response. Data in *(j)* are normalized as the fold of mock response.


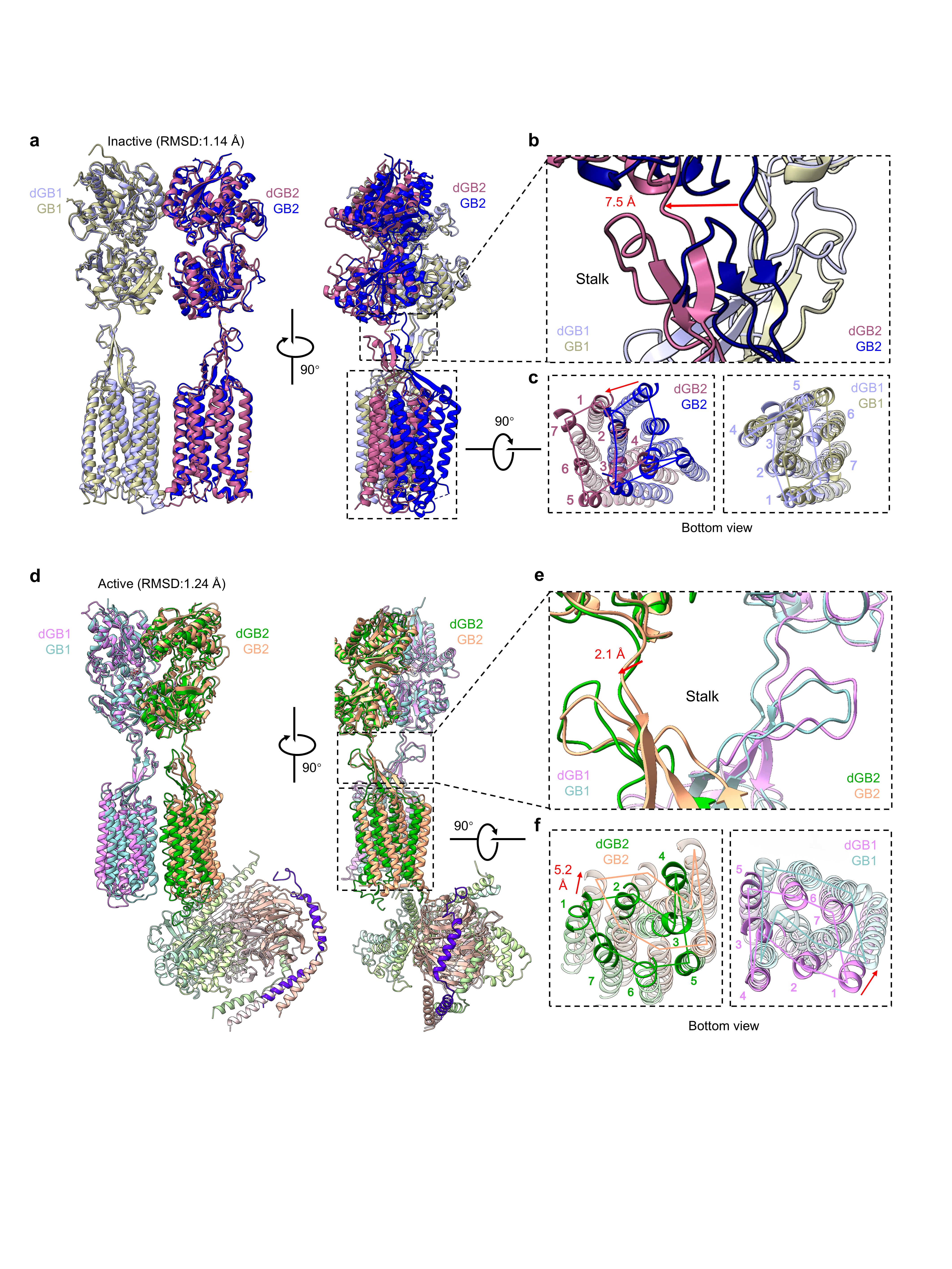


**Figure S6. Structural comparisons of the full-length *drosophila* and human GABA_B_ receptors in different states.**

**(a) and (d)** Structural comparisons of human and *drosophila* GABA_B_ receptors in inactive state *(a)***,** and in the active state complexed with heterotrimeric G proteins *(d)*. **(b–c) and (e–f)** Zoomed-in views comparing the changes in the stalk *(b and e)* and 7TM domains *(c and f)* between human and *drosophila* GABA_B_ receptors in different states.


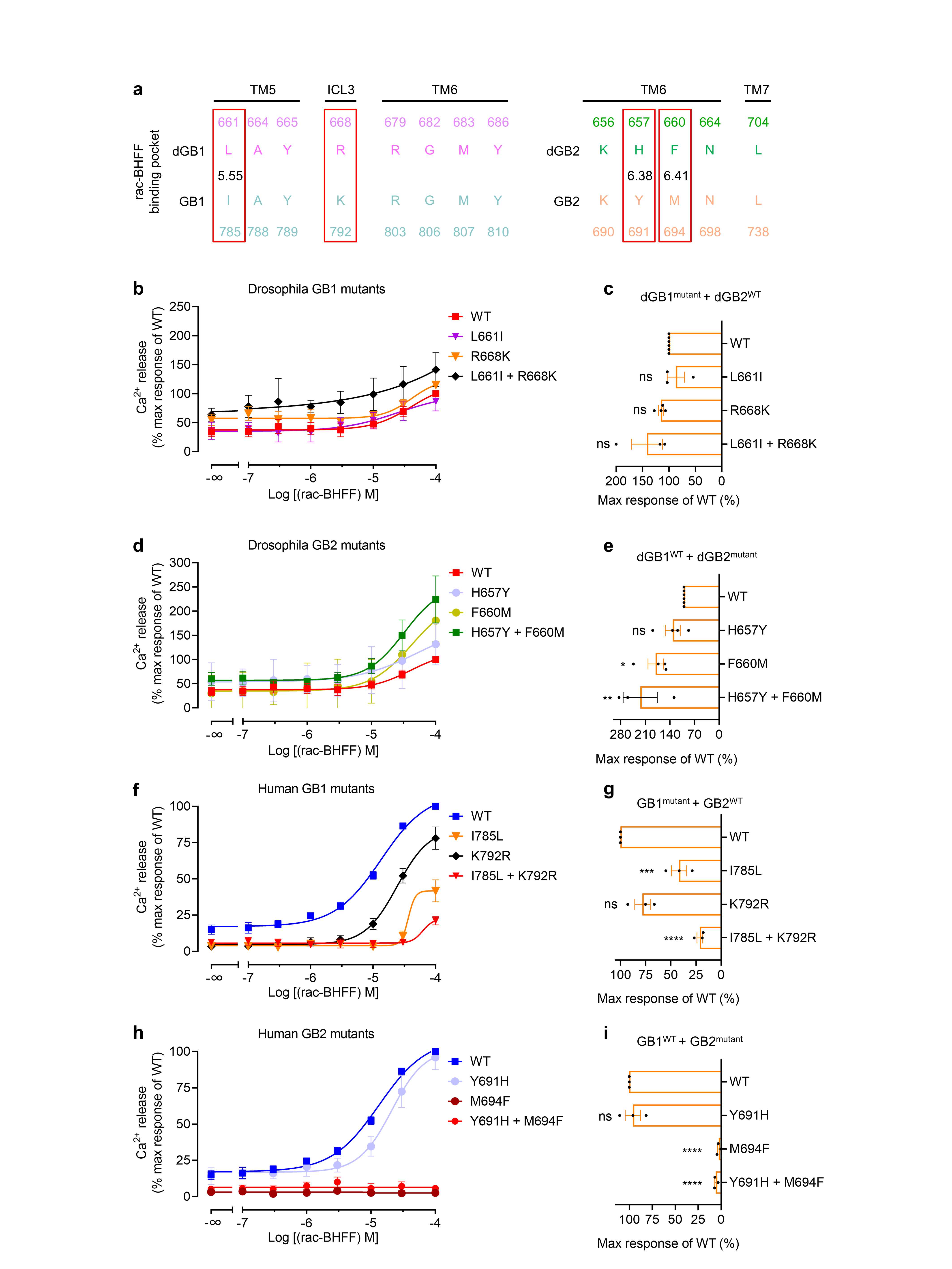


**Figure S7. Mutation analysis of residues in the PAM binding pocket of GABA_B_ receptors.**

**(a)** Amino acids sequences alignment of the residues in the PAM binding pocket of human and *drosophila* GABA_B_ receptors. The non-conserved residues were highlighted red boxes. **(b-i)** Intracellular calcium release induced by rac-BHFF in HEK293 cells co-transfected with the indicated constructs in the presence of EC_20_ of GABA. Data are normalized by wild-type GABA_B_ receptor response. Bars represent differences in calculated maximal efficacy (*E*_max_) for each mutant as a percentage of the maximum in wild-type. The indicated maximal efficacy is from the dose–response in wild-type GABA_B_ receptor and each mutant. Data in *(c)* and *(e)* correspond to *(b)* and *(d)*, data in *(g)* and *(i)* correspond to *(f)* and *(h)*, respectively. Data are mean ± SEM from at least three independent experiments, performed in technical duplicate and analyzed using one-way analysis of variance with Dunnett’s multiple comparison test to determine significance (compared with wild-type). Ns, not significant.


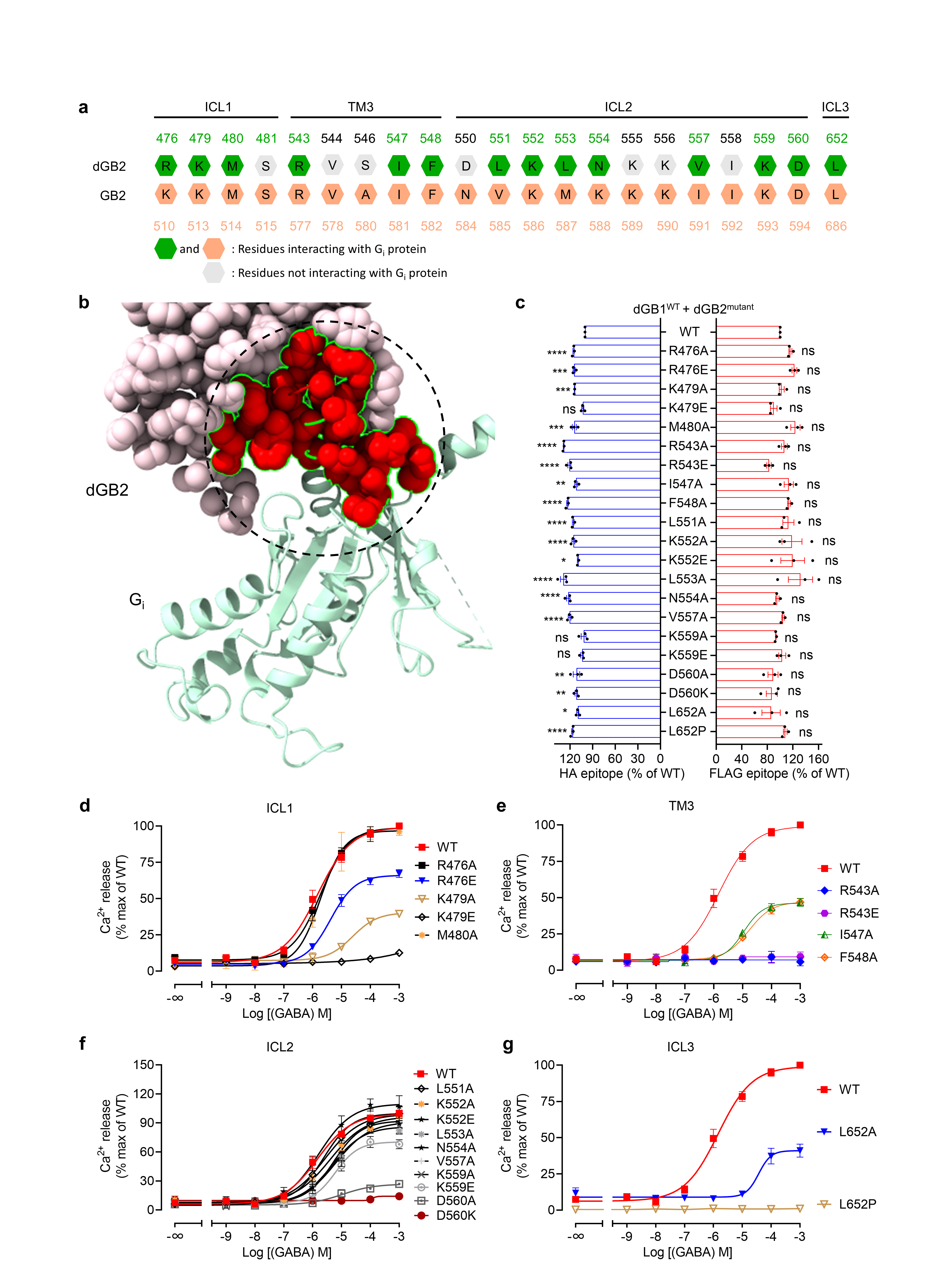


**Figure S8. Mutagenesis analyses of residues involved in the G protein binding pocket of *drosophila* GABA_B_ receptor.**

**(a)** Sequence alignments of residues in the G protein binding pocket of human and *drosophila* GB2 subunits. Residues in the interaction interface with G_i1_ of GB2 and dGB2 are highlighted in orange and green pentagons, respectively, while residues absent in the interface are highlighted in gray pentagon. **(b)** Sphere representation of the interaction interface between dGB2 (pink) and G_i1_ protein (light green). The interaction residues in dGB2 are highlighted in red sphere and black dotted circle. **(c)** ELISA measurement of the amount of indicated HA-tagged dGB1 and FLAG-tagged dGB2. HEK293 cells were transfected with wild-type dGB1 and dGB2 wild-type and mutants. Data are normalized by wild-type response. Data are mean ± SEM from at least three independent experiments, performed in technical duplicate and analyzed using one-way analysis of variance with Dunnett’s multiple comparison test to determine significance (compared with wild-type). ns: not significant. **(d–g)** Intracellular calcium release induced by GABA in HEK293 cells co-transfected with wild-type dGB1 and the indicated dGB2 mutants within ICL1 *(d)*, TM3 *(e)*, ICL2 *(f)* and ICL3 *(g)*. Data are normalized by wild-type response. Data are shown as means ± SEM of at least three biologically independent experiments.


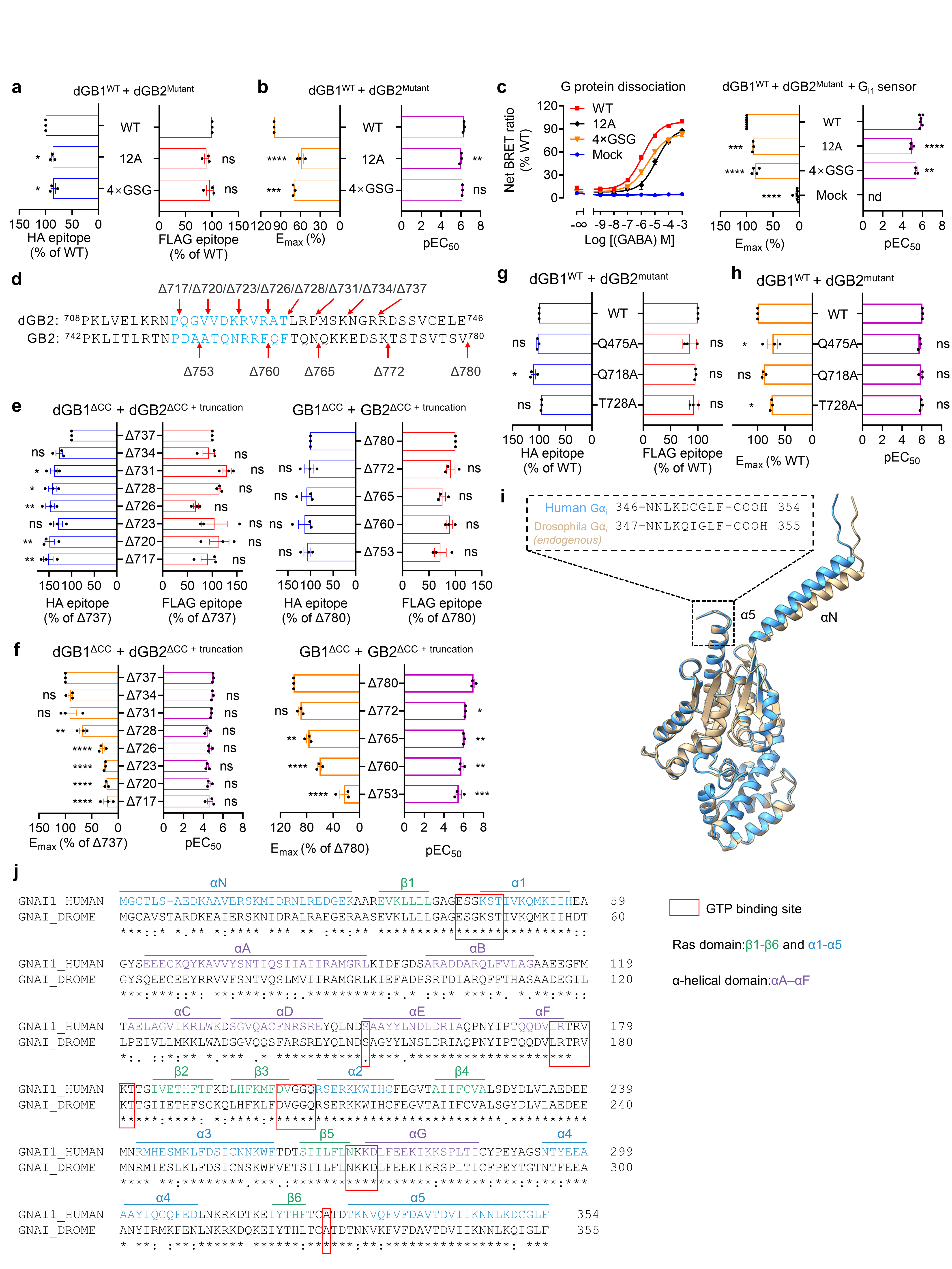


**Figure S9. The GB2 subunit C-terminus owns a similar effect as dGB2 in stabilizing G proteins.**

**(a) and (g)** ELISA measurement of the amount of HA-tagged dGB1 and FLAG-tagged dGB2 in HEK293 cells co-transfected with wild-type dGB1 and the indicated dGB2 mutants. **(b) and (h)** Intracellular calcium release induced by GABA in HEK293 cells co-transfected with wild-type dGB1 and the indicated dGB2 mutants. **(c)** BRET experiments for G protein disassociation analysis in HEK293 cells co-transfected with wild-type dGB1, the indicated dGB2 mutants and G protein sensor. **(d)** Sequence alignments of the proximal C-terminus truncations of dGB2 and GB2 subunits. The ordered C-terminus in dGB2 and the corresponding region in GB2 were highlighted in blue. The truncation sites are indicated with arrows. **(e)** ELISA measurement of the amount of HA-tagged dGB1 (left) or GB1 (right), and FLAG-tagged dGB2 (left) or GB2 (right) in HEK293 cells co-transfected with the indicated constructs. **(f)** Intracellular calcium release induced by GABA in HEK293 cells co-transfected with the indicated *drosophila* (left) and human (right) GABA_B_ receptor constructs. **(b), (c), (f) and (h)** Bars represent differences in calculated maximal efficacy (*E*_max_) and potency (pEC_50_) for each dGB2 mutant as a percentage of the maximum in wild-type. Data are shown as means ± SEM of at least three biologically independent experiments. Values in *(a), (e) and (g)* are mean ± SEM from at least three independent experiments, performed in technical duplicate. Data are normalized by Δ737, Δ780 or wild-type response as indicated, and analyzed using one-way analysis of variance with Dunnett’s multiple comparison test to determine significance (compared with Δ737, Δ780 or wild-type). ns, not significant. **(i)** Superimposed structures of G_i1_ proteins in *drosophila* and human (RMSD = 0.394 Å). The proximal sequences alignment of α5 Helix domain of G_i1_ proteins are present. Human G_i1_: from PDB 7EB2. *Drosophila* G_i1_: predicated by program Alphafold2. **(j)** Sequence alignment of *drosophila* and human Gi1 proteins with annotated key structural domains. The green (β1-6) and blue (α1-5) regions represent the Ras domain, the purple regions denote the α-helical domain, and red boxes highlight the GTP binding sites.


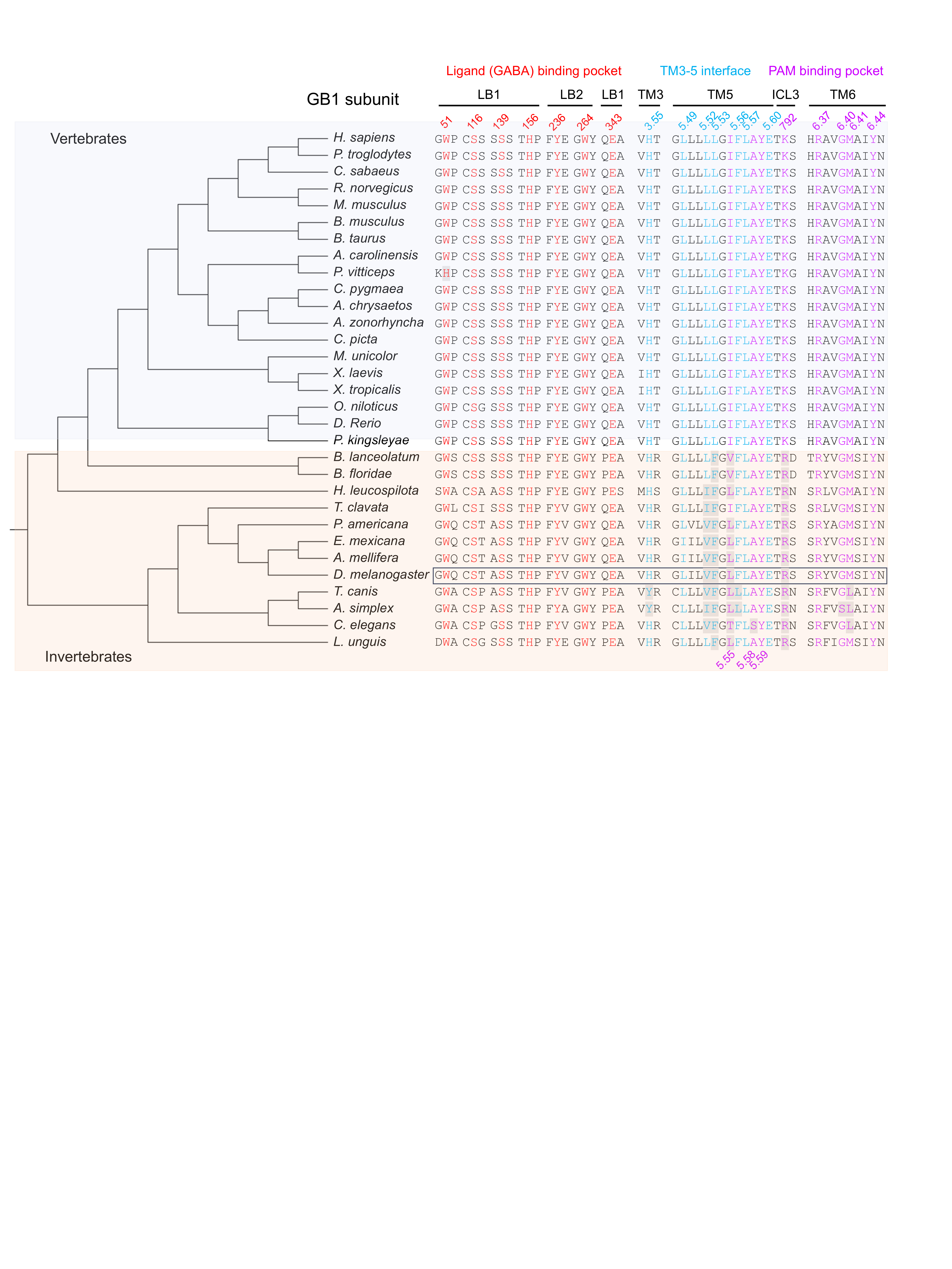


**Figure S10. Phylogenetic analysis of GB1 subunits during evolution.**

Sequences are from Universal Protein database (https://www.uniprot.org/). The alignment was generated with ClustalW. The construction of phylogenetic tree based on TimeTree (http:// www.timetree.org/home). Red denotes the ligand-binding pocket; blue labels critical amino acids at the TM3-5 interface in GB1; purple represents residues associated with the PAM binding pocket in GB1. Gray shading indicates non-conserved sites. The complete names of these species were shown in Table S7.


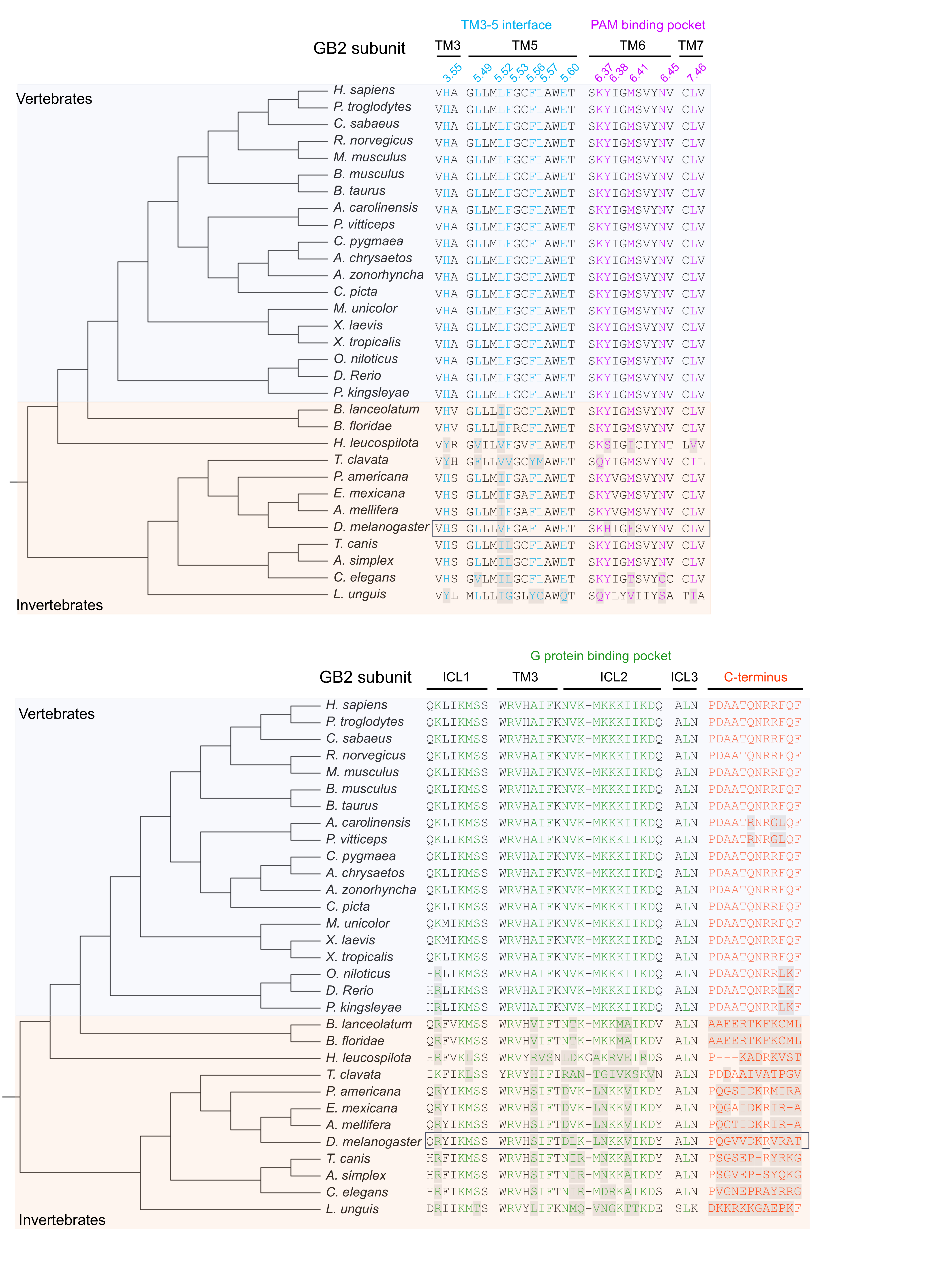


**Figure S11. Phylogenetic analysis of GB2 subunits during evolution.**

Sequences are from Universal Protein database (https://www.uniprot.org/). The alignment was generated with ClustalW. The construction of phylogenetic tree based on TimeTree (http:// www.timetree.org/home). Blue labels critical amino acids at the TM3-5 interface in GB2; purple represents residues associated with the PAM binding pocket in GB2; The green and orange colored C-terminus regions indicate the G protein-binding pocket. Gray shading indicates non-conserved sites. The complete names of these species were shown in Table S7.


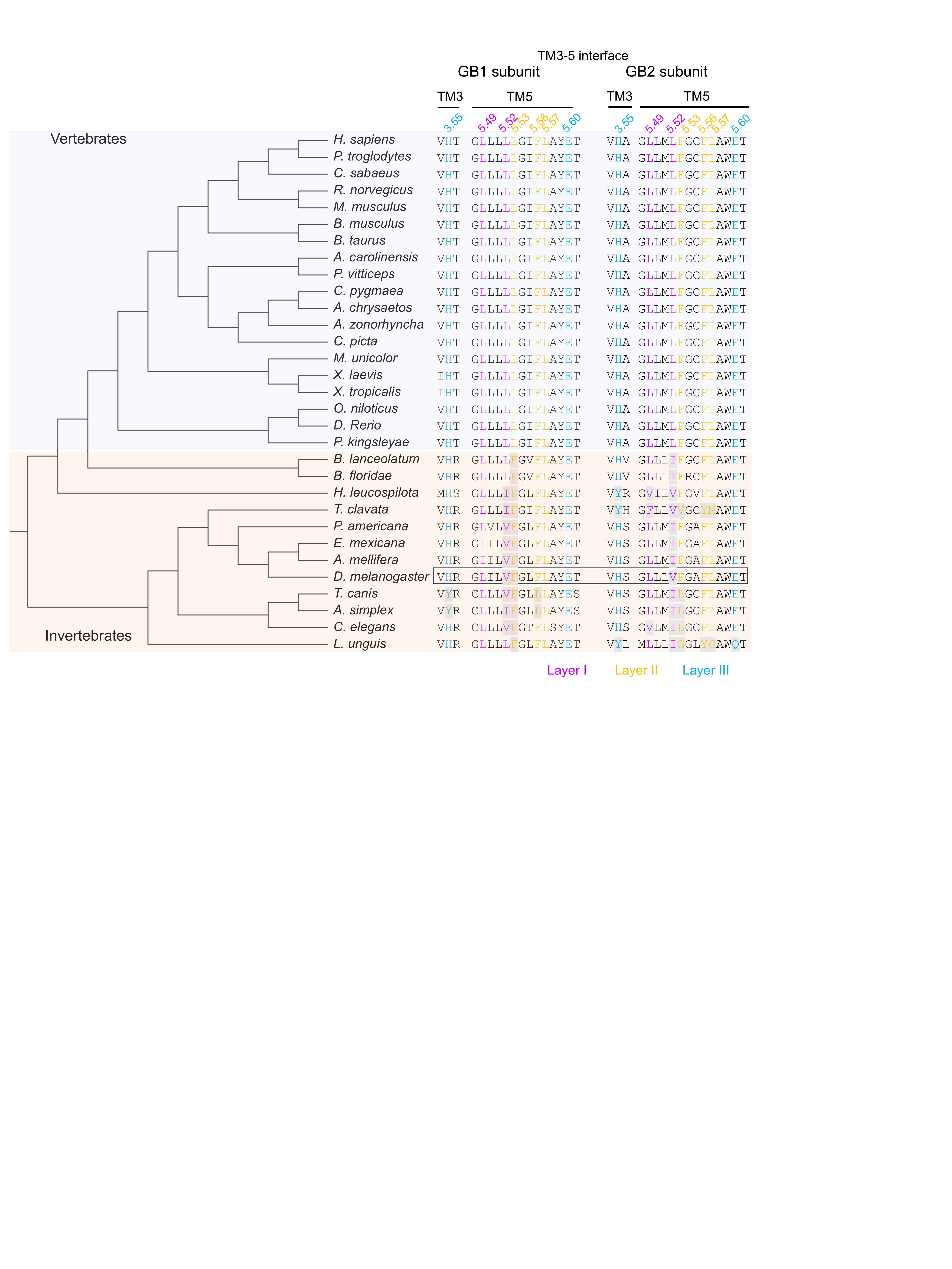


**Figure S12. Phylogenetic analysis of the layer I-III residues in the TM3-5 interface within GABA_B_ receptor heterodimer.**

The alignment was from Figure S10 and S11. Purple, yellow, and blue respectively label critical amino acids at the layer I-III of TM3-5 interface in GB1 and GB2. Gray shading indicates non-conserved sites. The amino acid sequences of *drosophila* GABA_B_ receptor are highlighted with black boxes. The complete names of these species were shown in Table S7.

**Table S1. Statistics of potency analysis of GABA or CGP54626 for the *drosophila* and human GABA_B_ receptors respectively.**

| Agonist | Receptor | pEC_50_ ± SEM | n | P value |
| --- | --- | --- | --- | --- |
| GABA | *Drosophila* GABA_B_ receptors | 6.45 ± 0.12 | 3 | 0.3087 |
|  | Human GABA_B_ receptors | 6.72 ± 0.20 | 3 |  |
| Antagonist | Receptor | pEC_50_ ± SEM | n | P value |
| CGP54626 | *Drosophila* GABA_B_ receptors | 5.44 ± 0.11 | 4 | <0.0001 |
|  | Human GABA_B_ receptors | 7.11 ± 0.07 | 4 |  |

Data are mean ± SEM from at least three independent experiments, performed in technical duplicate and analyzed using the unpaired t-test (two-tailed). Data are normalized by the max response respectively.

**Table S2. Effects of mutations on *drosophila* GABA_B_ receptors at the TM3-5 interface.**

| Group | Mutants | Basal IP_1_ accumulation  fold of mock  (mean ± SEM) ^a^ | n | P value | Expression (% WT) ^b^ | | | | | |
| --- | --- | --- | --- | --- | --- | --- | --- | --- | --- | --- |
|  |  |  |  |  | Anti-HA | P value | n | Anti-FLAG | P value | n |
| Mock | Mock | 1.00 ± 0.00 | 3 | 0.9897 | 2.18 ± 0.03 | <0.0001 | 4 | 6.99 ± 0.66 | <0.0001 | 3 |
| dGB1^WT^ + dGB2^WT^ | dGB1^WT^ | 1.07 ± 0.03 | 3 | - | 100.00 ± 0.00 | - | 4 | 100.00 ± 0.00 | - | 4 |
| I + WT | I: dGB1^V658L^ | 1.60 ± 0.17 | 3 | 0.0085 | 106.41 ± 6.46 | 0.8376 | 4 | 97.91± 9.41 | 0.9996 | 3 |
| II + WT | II: dGB1^F659L^ | 1.77 ± 0.06 | 3 | 0.0008 | 101.13 ± 4.49 | 0.9997 | 4 | 114.67 ± 2.18 | 0.1804 | 3 |
| III + WT | III: dGB1^H566A-E666A^ | 1.69 ± 0.10 | 3 | 0.0022 | 76.17 ± 1.46 | 0.0085 | 4 | 61.34 ± 3.24 | 0.0002 | 3 |
| I-II + WT | I-II: dGB1^V658L-F659L^ | 2.16 ± 0.08 | 3 | <0.0001 | 108.02 ± 7.11 | 0.6827 | 4 | 111.60 ± 2.30 | 0.3731 | 3 |
| I-II-III + WT | I-II-III: dGB1^V658L-F659L- H566A-E666A^ | 2.27 ± 0.10 | 3 | <0.0001 | 57.75 ± 6.26 | <0.0001 | 4 | 73.31 ± 8.90 | 0.0057 | 3 |

^a^ Data are normalized as the fold of mock response. ^b^ Data are normalized by the wild-type response. All data are shown means ± SEM from at least three independent experiments performed in technical duplicate and analyzed using one-way ANOVA with Dunnett’s multiple comparison test to determine significance compared with wild-type.

**Table S3. Effects of mutations on GABA_B_ receptors at PAM binding pocket (*drosophila* and human GABA_B_ receptors respectively)**

| Mutants | | *E*_max_ ± SEM | n | P value |
| --- | --- | --- | --- | --- |
| dGB1^WT^ | dGB2^WT^ | 100.00 | 6 | - |
| dGB1^L661I-R668K^ | dGB2^H657Y-F660M^ | 240.50 ± 27.05 | 5 | 0.0003 |
| dGB1^L661I-R668K^ | dGB2^WT^ | 141.44 ± 29.33 | 3 | 0.0841 |
| dGB1^R668K^ | dGB2^WT^ | 115.10 ± 4.51 | 4 | 0.6853 |
| dGB1^L661I^ | dGB2^WT^ | 86.68 ± 16.43 | 3 | 0.8008 |
| dGB1^WT^ | dGB2^H657Y-F660M^ | 224.28 ± 48.54 | 3 | 0.0043 |
| dGB1^WT^ | dGB2^H657Y^ | 131.62 ± 21.33 | 4 | 0.5876 |
| dGB1^WT^ | dGB2^F660M^ | 180.53 ± 21.67 | 4 | 0.0379 |
| Mutants | | *E*_max_ ± SEM | n | P value |
| GB1^WT^ | GB2^WT^ | 100.00 | 3 | - |
| GB1^I785L-K792R^ | GB2^Y691H-M694F^ | 5.32 ± 0.81 | 3 | <0.0001 |
| GB1^I785L-K792R^ | GB2^WT^ | 21.10 ± 2.83 | 3 | <0.0001 |
| GB1^I785L^ | GB2^WT^ | 41.75 ± 7.54 | 3 | 0.0002 |
| GB1^K792R^ | GB2^WT^ | 78.10 ± 7.75 | 3 | 0.0587 |
| GB1^WT^ | GB2^Y691H-M694F^ | 5.65 ± 1.23 | 3 | <0.0001 |
| GB1^WT^ | GB2^Y691H^ | 95.97 ± 8.31 | 3 | 0.8423 |
| GB1^WT^ | GB2^M694F^ | 2.62 ± 1.23 | 3 | <0.0001 |

Data are mean ± SEM from at least three independent experiments, performed in technical duplicate and analyzed using one-way ANOVA with Dunnett’s multiple comparison test to determine significance compared with wild-type respectively.

**Table S4. Effects of mutations on *drosophila* GABA_B_ receptors at the G protein binding pocket.**

| **Mutants** | ***E*_max_ ± SEM** | **n** | **P value** | **pEC_50_ ± SEM** | **n** | **P value** | **Expression (% WT)** | | | | | |
| --- | --- | --- | --- | --- | --- | --- | --- | --- | --- | --- | --- | --- |
|  |  |  |  |  |  |  | **Anti-HA** | **n** | **P value** | **Anti-FLAG** | **P value** | **n** |
| dGB2^WT^ | 100.00 | 4 | - | 5.84 ± 0.17 | 4 | - | 100.00 | 3 | - | 100.00 | 3 | - |
| dGB2^R476A^ | 100.32 ± 3.74 | 4 | >0.9999 | 5.73 ± 0.13 | 4 | 0.9993 | 115.22 ± 0.88 | 3 | <0.0001 | 116.11 ± 2.40 | 3 | 0.8871 |
| dGB2^R476E^ | 67.26 ± 2.70 | 4 | <0.0001 | 5.18 ± 0.18 | 4 | 0.0774 | 114.38 ± 1.37 | 3 | 0.0001 | 122.36 ± 3.78 | 3 | 0.5290 |
| dGB2^K479A^ | 39.49 ± 0.41 | 4 | <0.0001 | 4.62 ± 0.20 | 4 | <0.0001 | 113.93 ± 0.22 | 3 | 0.0002 | 102.56 ± 3.88 | 3 | 0.9997 |
| dGB2^K479E^ | 12.59 ± 1.63 | 4 | <0.0001 | - | 4 | nd | 102.87 ± 1.43 | 3 | 0.9857 | 90.08 ± 5.14 | 3 | 0.9951 |
| dGB2^M480A^ | 96.01 ± 2.49 | 3 | 0.9991 | 5.55 ± 0.19 | 4 | 0.8934 | 114.08 ± 2.66 | 3 | 0.0002 | 123.92 ± 7.18 | 3 | 0.4395 |
| dGB2^R543A^ | 5.75 ± 2.75 | 4 | <0.0001 | - | 4 | nd | 128.32 ± 0.52 | 3 | <0.0001 | 106.72 ± 4.48 | 3 | 0.9992 |
| dGB2^R543E^ | 9.33 ± 3.20 | 4 | <0.0001 | - | 4 | nd | 120.77 ± 2.01 | 3 | <0.0001 | 82.53 ± 3.49 | 3 | 0.8217 |
| dGB2^I547A^ | 46.78 ± 2.70 | 4 | <0.0001 | 4.94 ± 0.09 | 4 | 0.0051 | 111.71 ± 1.84 | 3 | 0.0025 | 113.53 ± 7.17 | 3 | 0.9676 |
| dGB2^F548A^ | 46.86 ± 2.38 | 4 | <0.0001 | 4.78 ± 0.05 | 4 | 0.0006 | 123.25 ± 1.06 | 3 | <0.0001 | 113.90 ± 2.26 | 3 | 0.9594 |
| dGB2^L551A^ | 99.59 ± 7.51 | 4 | >0.9999 | 5.78 ± 0.14 | 4 | 0.9996 | 115.69 ± 1.09 | 3 | <0.0001 | 112.97 ± 8.68 | 3 | 0.9775 |
| dGB2^K552A^ | 97.75 ± 3.74 | 4 | 0.9995 | 5.31 ± 0.16 | 4 | 0.2476 | 115.50 ± 1.93 | 3 | <0.0001 | 118.54 ± 15.61 | 3 | 0.7624 |
| dGB2^K552E^ | 89.30 ± 2.18 | 4 | 0.6286 | 5.29 ± 0.14 | 4 | 0.2134 | 109.44 ± 0.60 | 3 | 0.0237 | 119.66 ± 18.57 | 3 | 0.6950 |
| dGB2^L553A^ | 81.80 ± 3.54 | 4 | 0.0636 | 5.27 ± 0.10 | 4 | 0.1769 | 128.65 ± 3.83 | 3 | <0.0001 | 131.76 ± 18.93 | 3 | 0.1341 |
| dGB2^N554A^ | 107.08 ± 11.21 | 4 | 0.9668 | 5.58 ± 0.17 | 4 | 0.9491 | 122.19 ± 2.22 | 3 | <0.0001 | 95.59 ± 2.69 | 3 | 0.9995 |
| dGB2^V557A^ | 99.52 ± 7.11 | 4 | >0.9999 | 5.38 ± 0.16 | 4 | 0.4016 | 120.38 ± 1.83 | 3 | <0.0001 | 104.60 ± 1.90 | 3 | 0.9995 |
| dGB2^K559A^ | 86.99 ± 7.63 | 3 | 0.4675 | 5.19 ± 0.07 | 4 | 0.0876 | 101.80 ± 3.31 | 3 | 0.9991 | 93.27 ± 0.63 | 3 | 0.9992 |
| dGB2^K559E^ | 67.96 ± 4.67 | 4 | <0.0001 | 5.24 ± 0.11 | 4 | 0.1358 | 103.94 ± 1.65 | 3 | 0.8478 | 103.27 ± 5.47 | 3 | 0.9997 |
| dGB2^D560A^ | 26.86 ± 3.12 | 4 | <0.0001 | 4.48 ± 0.36 | 4 | <0.0001 | 111.27 ± 4.32 | 3 | 0.0039 | 88.93 ± 7.83 | 3 | 0.9899 |
| dGB2^D560K^ | 14.16 ± 2.10 | 4 | <0.0001 | - | 4 | nd | 111.55 ± 1.73 | 3 | 0.0029 | 86.98 ± 8.64 | 3 | 0.9768 |
| dGB2^L652A^ | 41.02 ± 4.48 | 4 | <0.0001 | 4.33 ± 0.25 | 3 | <0.0001 | 109.55 ± 1.39 | 3 | 0.0214 | 85.85 ± 14.32 | 3 | 0.9535 |
| dGB2^L652P^ | 1.01 ± 0.25 | 3 | <0.0001 | - | 3 | nd | 116.79 ± 1.10 | 3 | <0.0001 | 107.99 ± 2.90 | 3 | 0.9990 |

Data are normalized by wild-type response. Values are shown as means ± SEM of at least three biologically independent experiments, performed in technical triplicate, and analyzed using one-way ANOVA with Dunnett’s multiple comparison test to determine significance compared with wild-type. Nd, not determined. which refers to can’t be established over the tested concentration range, such that an *E*_max_ was not reached and therefore span could not be calculated.

**Table S5. Effects of truncations and mutations on GABAB receptors at the C-terminal of GB2 subunit.**

| Mutants | *E*_max_ ± SEM ^a^ | n | P value | pEC_50_ ± SEM | n | P value | Expression (% WT) ^a^ | | | | |
| --- | --- | --- | --- | --- | --- | --- | --- | --- | --- | --- | --- |
|  |  |  |  |  |  |  | Anti-HA | P value | Anti-FLAG | P value | n |
| dGB2^WT^ | 100.00 ± 0.00 | 3 | - | 6.29 ± 0.05 | 3 | - | 100.00 ± 0.00 | - | 100.00 ± 0.00 | - | 3 |
| 12A | 59.25 ± 3.57 | 3 | <0.0001 | 5.99 ± 0.06 | 3 | 0.0059 | 87.04 ± 2.51 | 0.0323 | 89.59 ± 4.33 | 0.2334 | 3 |
| 4 × GSG | 70.00 ± 1.08 | 3 | 0.0001 | 6.15 ± 0.02 | 3 | 0.1056 | 85.70 ± 4.26 | 0.0216 | 95.98 ± 6.17 | 0.7495 | 3 |
| dGB2^Q475A^ | 73.04 ± 9.37 | 3 | 0.0110 | 5.78 ± 0.08 | 3 | 0.1023 | 102.30 ± 1.13 | 0.7997 | 84.76 ± 7.10 | 0.0808 | 3 |
| dGB2^Q718A^ | 88.85 ± 2.10 | 3 | 0.3068 | 5.92 ± 0.12 | 3 | 0.5242 | 111.24 ± 4.18 | 0.0164 | 95.55 ± 0.72 | 0.7996 | 3 |
| dGB2^T728A^ | 74.72 ± 1.27 | 3 | 0.0154 | 5.94 ± 0.08 | 3 | 0.6174 | 95.44 ± 0.50 | 0.3720 | 92.51 ± 4.49 | 0.4896 | 3 |
| Truncations | *E*_max_ ± SEM ^b^ | n | P value | pEC_50_ ± SEM | n | P value | Expression (% dGB1^ΔCC^) | P value | Expression (% dGB2^Δ737^) ^b^ | P value | n |
| dGB2^Δ737^ | 100.00 ± 0.00 | 3 | - | 4.99 ± 0.03 | 3 | - | 100.00 ± 0.00 | - | 100.00 ± 0.00 | - | 3 |
| dGB2^Δ734^ | 90.53 ± 4.21 | 3 | 0.7762 | 4.93 ± 0.06 | 3 | 0.9996 | 127.10 ± 7.41 | 0.1890 | 92.94 ± 11.69 | 0.9994 | 3 |
| dGB2^Δ731^ | 91.75 ± 12.70 | 3 | 0.8604 | 4.81 ± 0.03 | 3 | 0.8893 | 138.79 ± 8.51 | 0.0326 | 130.03 ± 8.42 | 0.4932 | 3 |
| dGB2^Δ728^ | 67.58 ± 5.53 | 3 | 0.0077 | 4.44 ± 0.17 | 3 | 0.0628 | 142.74 ± 8.65 | 0.0172 | 114.43 ± 3.01 | 0.9554 | 3 |
| dGB2^Δ726^ | 30.26 ± 4.08 | 3 | <0.0001 | 4.67 ± 0.14 | 3 | 0.4491 | 147.76 ± 9.41 | 0.0075 | 67.33 ± 5.14 | 0.4109 | 3 |
| dGB2^Δ723^ | 24.73 ± 1.05 | 3 | <0.0001 | 4.43 ± 0.11 | 3 | 0.0595 | 129.93 ± 10.01 | 0.1268 | 104.65 ± 25.56 | 0.9997 | 3 |
| dGB2^Δ720^ | 22.00 ± 2.04 | 3 | <0.0001 | 4.62 ± 0.15 | 3 | 0.3256 | 148.55 ± 10.28 | 0.0066 | 114.32 ± 19.68 | 0.9570 | 3 |
| dGB2^Δ717^ | 20.47 ± 7.11 | 3 | <0.0001 | 4.71 ± 0.27 | 3 | 0.5951 | 151.68 ± 10.61 | 0.0040 | 91.59 ± 14.22 | 0.9974 | 3 |
| Truncations | *E*_max_ ± SEM ^c^ | n | P value | pEC_50_ ± SEM | n | P value | Expression (% GB1^ΔCC^) | P value | Expression (% GB2^Δ780^) ^c^ | P value | n |
| GB2^Δ780^ | 100.00 ± 0.00 | 3 | - | 6.99 ± 0.13 | 3 | - | 100.00 ± 0.00 | - | 100.00 ± 0.00 | - | 3 |
| GB2^Δ772^ | 89.57 ± 2.54 | 3 | 0.1724 | 6.17 ± 0.02 | 3 | 0.0161 | 103.09 ± 10.82 | 0.9979 | 91.29 ± 7.91 | 0.7973 | 3 |
| GB2^Δ765^ | 77.62 ± 2.86 | 3 | 0.0036 | 6.02 ± 0.05 | 3 | 0.0055 | 108.63 ± 10.95 | 0.9205 | 75.47 ± 5.88 | 0.1012 | 3 |
| GB2^Δ760^ | 60.71 ± 2.63 | 3 | <0.0001 | 5.78 ± 0.16 | 3 | 0.0012 | 113.18 ± 13.37 | 0.7467 | 89.25 ± 5.48 | 0.6703 | 3 |
| GB2^Δ753^ | 23.67 ± 6.26 | 3 | <0.0001 | 5.50 ± 0.29 | 3 | 0.0002 | 106.62 ±7.74 | 0.9665 | 71.52 ±11.06 | 0.0534 | 3 |

Data are normalized by wild-type ^a^, Δ737 ^b^ or Δ780 ^c^ response respectively, and analyzed using one-way ANOVA with Dunnett’s multiple comparison test to determine significance compared with wild-type, Δ737 or Δ780 respectively.

**Table S6. BRET experiments for G protein disassociation on *drosophila* GABA_B_** **receptors at the C-terminal of GB2 subunit.**

| Mutants | *E*_max_ ± SEM | n | P value | pEC_50_ ± SEM | n | P value |
| --- | --- | --- | --- | --- | --- | --- |
| Mock | 5.03 ± 1.35 | 7 | <0.0001 | - | 7 | nd |
| dGB2^WT^ | 100.00 ± 0.00 | 7 | - | 5.86 ± 0.06 | 6 | - |
| 12A | 87.78 ± 0.57 | 3 | 0.0002 | 4.91 ± 0.11 | 3 | <0.0001 |
| 4 × GSG | 83.43 ± 3.19 | 4 | <0.0001 | 5.38 ± 0.09 | 3 | 0.0034 |

Data are normalized by wild-type response. Values are shown as means ± SEM of at least three biologically independent experiments, performed in technical triplicate, and analyzed using one-way ANOVA with Dunnett’s multiple comparison test to determine significance compared with wild-type. Nd, not determined. which refers to can’t be established over the tested concentration range, such that an *E*_max_ was not reached and therefore span could not be calculated.

**Table S7. Table of species full names and abbreviated forms**

| Abbreviation | Complete Name |
| --- | --- |
| *H. sapiens* | *Homo sapiens* |
| *P. troglodytes* | *Pan troglodytes* |
| *C. sabaeus* | *Chlorocebus sabaeus* |
| *M. musculus* | *Mus musculus* |
| *R. norvegicus* | *Rattus norvegicus* |
| *B. taurus* | *Bos taurus* |
| *B. musculus* | *Balaenoptera musculus* |
| *A. chrysaetos* | *Aquila chrysaetos* |
| *C. pygmaea* | *Calidris pygmaea* |
| *A. zonorhyncha* | *Anas zonorhyncha* |
| *P. vitticeps* | *Pogona vitticeps* |
| *A. carolinensis* | *Anolis carolinensis* |
| *C. picta* | *Chrysemys picta* |
| *X. laevis* | *Xenopus laevis* |
| *X. tropicalis* | *Xenopus tropicalis* |
| *M. unicolor* | *Microcaecilia unicolor* |
| *P. kingsleyae* | *Paramormyrops kingsleyae* |
| *D. rerio* | *Danio rerio* |
| *O. niloticus* | *Oreochromis niloticus* |
| *B. lanceolatum* | *Branchiostoma lanceolatum* |
| *B. floridae* | *Branchiostoma floridae* |
| *H. leucospilota* | *Holothuria leucospilota* |
| *D. melanogaster* | *Drosophila melanogaster* |
| *P. americana* | *Periplaneta americana* |
| *E. mexicana* | *Eufriesea mexicana* |
| *T. clavata* | *Trichonephila clavata* |
| *A. mellifera* | *Apis mellifera* |
| *C. elegans* | *Caenorhabditis elegans* |
| *T. canis* | *Toxocara canis* |
| *A. simplex* | *Anisakis simplex* |
| *L. unguis* | *Lingula unguis* |

**Table S8. Comparison of structures of drosophila and human GABA_B_ receptors.**

| State | PDB | Resolution (Å) | Assembly | Constructs | ligands | Binding pocket | Ref. |
| --- | --- | --- | --- | --- | --- | --- | --- |
| Active  state |  | 3.3 | dGB1+dGB2+ G_i1_Gβ_1_γ_2_ | dGB1:29-800  dGB2:24-790 | GABA (AGO) | **GABA**: dGB1 (W51, S116, S139, H156, Y236, W264, E343) | This study |
|  | 4MS4 | 1.90 | hGB1b-hGB2  VFT | hGB1b:48-459  hGB2:1-466 | Baclofen (AGO) | **Baclofen/GABA**: GB1b (W65, S130, S153, H170, Y250, W278, E349) | Geng et al. (2013)  **(X-ray)** |
|  | 4MS3 | 2.50 |  |  | GABA (AGO) |  |  |
|  | 6UOA | 6.3 | hGB1a-GB2 | hGB1a:165-920  hGB2:42-821 | SKF97541 (AGO) | **SKF97541**: GB1a (W182, S247, S270, H287, Y367, W395, E466); **GS39783**: GB1a (Y789^5.59^, K792^ICL3^, M807^6.41^, Y810^6.44^); GB2 (M694^6.41^, Y697^6.44^, N698^6.45^) | Shaye et al.  (2020) |
|  | 6UO9 | 4.8 |  |  |  |  |  |
|  | 6UO8 | 3.6 |  |  | SKF97541(AGO) + GS39783 (PAM) |  |  |
|  | 7C7Q | 3.0 | hGB1a-GB2 +  hGi + scFv16 | hGB1a:15-860  hGB2:42-780 | Baclofen (AGO) +  rac-BHFF (PAM) | **Baclofen**: GB1a (W182, S247, S270, H287, Y367, W395, E466);  **rac-BHFF**: GB1a (I785^5.56^, A788^5.58^, Y789^5.59^, K792^ICL3^, R803^6.37^, G806^6.40^, M807^6.41^, Y810^6.44^); GB2 (K690^6.37^, Y691^6.38^, M694^6.41^, N698^6.45^, L738^7.46^) | Mao et al. (2020) |
|  | 7EB2 | 3.5 |  | hGB1a:15-919  hGB2:42-819 |  |  | Shen et al. (2021) |
|  | 7CA3 | 4.5 | hGB1a-GB2 | hGB1a:165-900  hGB2:1-787 | GABA (AGO) +  rac-BHFF (PAM) | **GABA**: GB1a (W182, S247, S270, H287, Y367, W395, E466);  **rac-BHFF**: GB1a (I785^5.56^, A788^5.58^, Y789^5.59^, K792^ICL3^, R803^6.37^, G806^6.40^, M807^6.41^, Y810^6.44^); GB2 (K690^6.37^, Y691^6.38^, M694^6.41^, N698^6.45^, L738^7.46^) | Kim et al. (2020) |

| State | PDB | Resolution (Å) | Assembly | Constructs | ligands | Binding pocket | Ref. |
| --- | --- | --- | --- | --- | --- | --- | --- |
|  |  | 3.52 | dGB1+dGB2 | dGB1:29-800  dGB2:24-790 | CGP54626 (ANT) | **CGP54626**: dGB1 (W51, S116, S139, H156, W264, E343) | This study |
| Inactive  state | 4MQE | 2.35 | hGB1b-hGB2  VFT | hGB1b:48-459  hGB2:1-466 | Apo | / | Geng et al. (2013)  **(X-ray)** |
|  | 4MR7 | 2.15 |  |  | CGP54626 (ANT) | **CGP54626/ SCH50911**: GB1b (W65, S130, S153, H170, W278, E349) |  |
|  | 4MR9 | 2.35 |  |  | SCH50911 (ANT) |  |  |
|  | 4MS1 | 2.25 |  |  | CGP46381 (ANT) | **CGP46381/CGP35348/Saclofen/ Phaclofen**: GB1b (W65, S130, S153, H170, E349) |  |
|  | 4MR8 | 2.15 |  |  | CGP35348 (ANT) |  |  |
|  | 4MQF | 2.22 |  |  | Saclofen (ANT) |  |  |
|  | 4MRM | 2.86 |  |  | Phaclofen (ANT) |  |  |
|  | 6VJM | 4.0 | hGB1a-hGB2 | hGB1a:165-920  hGB2:42-821 | Apo | / | Shaye et al. (2020) |
|  | 6W2X | 3.6 | hGB1b-hGB2 | hGB1b:30-844  hGB2:41-941 | CGP55845 (ANT) | **CGP55845**: GB1b (W65, S130, S153, H170, W278, E349) | Papasergi-Scott et al. (2020) |
|  | 6W2Y | 3.2 | hGB1b-hGB1b | hGB1b:30-844 |  |  |  |
|  | 6WIV | 3.3 | hGB1b-hGB2 | hGB1b:1-802  hGB2:1-819 | Apo | / | Park et al. (2020) |
|  | 7C7S | 2.9 | hGB1a-hGB2 | hGB1b:1-802  hGB2:1-819 | CGP54626 (ANT) | **CGP54626**: GB1a (W182, S247, S270, H287, W395, E466) | Mao et al. (2020) |
|  | 7CUM | 3.5 | hGB1a-hGB2 | hGB1a:165-900  hGB2:1-787 | CGP54626 (ANT) + CLH304a (NAM) |  | Kim et al. (2020) |
|  | 7CA5 | 7.6 |  |  | Apo | / |  |

| State | PDB | Resolution (Å) | Assembly | Constructs | ligands | Binding pocket | Ref. |
| --- | --- | --- | --- | --- | --- | --- | --- |
|  | 5X9X | / | dGB1-dGB2  coiled coil | dGB1:755-796 dGB2:741-780 | / | / | Zhang et al. (2014)  **(NMR)** |
|  | 4PAS | 1.6 | hGB1-hGB2  coiled coil | hGB1:762-802 hGB2:779-819 | / | / | Burmakina et al. (2014)  **(X-ray)** |
|  | 4F11 | 2.4 | hGB2 VFT | hGB2:1-466 | / | / | Geng et al. (2012)  **(X-ray)** |
|  | 4F12 | 3.0 |  |  | / | / |  |
